# Hydrogel Films with Impact Resistance by Sacrificial Micelle‐Assisted‐Alignment

**DOI:** 10.1002/advs.202409287

**Published:** 2024-10-07

**Authors:** Jingxian Zhang, Xiaowen Shi, Zhongtao Zhao, Manya Wang, Hongbing Deng, Yumin Du

**Affiliations:** ^1^ School of Resource and Environmental Science Hubei Engineering Center of Natural Polymers‐Based Medical Materials Hubei Biomass‐Resource Chemistry and Environmental Biotechnology Key Laboratory Hubei International Scientific and Technological Cooperation Base of Sustainable Resource and Energy Wuhan University Wuhan 430079 China

**Keywords:** chitosan, hierarchical designs, hydrogels, impact resistance, lobster underbelly, micelles

## Abstract

Various strategies are developed to engineer aligned hierarchical architectures in polymer hydrogels for enhanced mechanical performance. However, chain alignment remains impeded by the presence of hydrogen bonds between adjacent chains. Herein, a facile sacrificial micelle‐assisted‐alignment strategy is proposed, leading to well‐aligned, strong and tough pure chitosan hydrogels. The sacrificial sodium dodecyl sulfate micelles electrostatically interact with the protonated chitosan chains, enabling chain sliding and alignment under uniaxial forces. Subsequently, sacrificial micelles can be easily removed via NaOH treatment, causing the reforming of H‐bond in the chain networks. The strength of the pure chitosan hydrogels increases 140‐fold, reaching 58.9 ± 3.4 MPa; the modulus increases 595‐fold, reaching 226.4 ± 42.8 MPa. After drying‐rehydration, the strength and modulus further rise to 70.3 ± 2.4 and 403.5 ± 76.3 MPa, marking a significant advancement in high‐strength pure chitosan hydrogel films. Furthermore, the designed multiscale architectures involving enhanced crystallinity, well‐aligned fibers, strong interfaces, robust multilayer Bouligand assembly contribute to the exact replica of lobster underbelly with impact resistance up to 6.8 ± 1.0 kJ m^−1^. This work presents a promising strategy for strong, tough, stiff and impact‐resistant polymer hydrogels via well‐aligned hierarchical design.

## Graphical abstract

A sacrificial micelle‐assisted‐alignment strategy is proposed, leading to well‐aligned, strong pure chitosan hydrogels. The strength and modulus of chitosan hydrogels reach 70.3 ± 2.4 and 403.5 ± 76.3 MPa, marking a significant advancement in high‐strength pure chitosan hydrogel films. Furthermore, the designed replica of lobster underbelly shows impact resistance up to 6.8 ± 1.0 kJ m^−1^.

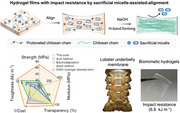

## 1 Introduction

High‐performance polymer hydrogels, featuring on‐demand functionalities, hold significant potential in tissue engineering, flexible electronics and impact‐resistant materials.^[^ ^1^ ^]^ Currently, promising strategies including freeze‐casting,^[^ ^2^, ^3^ ^]^ mechanical training,^[^ ^4^ ^]^ electrospinning,^[^ ^5^ ^]^ drying in confined condition,^[^ ^6^ ^]^ pre‐stretching^[^ ^7^, ^8^ ^]^ have been developed to create hydrogels with aligned architectures and hierarchical superstructures across multiple length scales, so as to enhance its mechanical properties (i.e., strength, toughness, modulus, impact resistance). However, due to chain's entanglement and rigidity, polymer chains are hard to align owing to the formation of H‐bonds between adjacent chains, resulting in low enhancement in mechanical strength. Therefore, it is plausible to deduce that adjusting the sequence of molecular chain alignment and H‐bond formation can further enhance mechanical properties. Given that aligned chains can induce more crystallites owing to chain proximity,^[^ ^7^ ^]^ we aim to effectively align polymer chains prior to H‐bond formation, thereby potentially enhancing the crystallinity and the mechanical properties.

**Figure 1:**
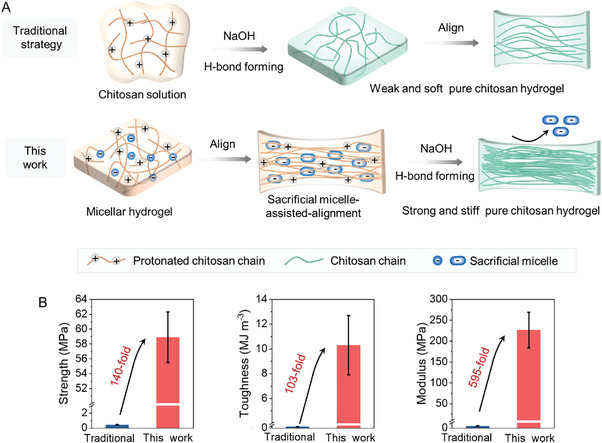
A) Design of strong, tough, stiff, anisotropic chitosan hydrogels via the sacrificial micelle‐assisted‐alignment strategy and comparison with the traditional strategy. B) The enhancement in strength, toughness and modulus of pure chitosan hydrogels prepared by the sacrificial micelle‐assisted‐alignment strategy.

Herein, we proposed a sacrificial micelle‐assisted‐alignment strategy to create robust hydrogels with aligned hierarchical structures (**Figure** **1A**). In traditional strategy,^[^ ^9^, ^10^, ^11^, ^12^, ^13^ ^]^ anisotropic chitosan hydrogels are formed by NaOH neutralization and then alignment. Due to the existence of H‐bonds, chains cannot effectively align, leading to weak and soft hydrogels. In this work, we sequentially disrupt H‐bonds, align the polymer chains, and then reconstruct the H‐bonds, resulting in strong and tough anisotropic chitosan hydrogels. Specifically, we enlist anionic sodium dodecyl sulfate (SDS) micelles to bind the protonated chitosan chains through strong electrostatic attractions, forming a micellar Chit‐H^+^/SDS hydrogel. SDS micelles weaken the mutual electrostatic repulsion between protonated chitosan chains, enabling closer chain spacing. Besides, micellar hydrogels, promising candidates for stretchable/aligned hydrogels, can effectively dissipate energy through shape deformation of micelles,^[^ ^14^, ^15^, ^16^ ^]^ and improve network orientation.^[^ ^17^ ^]^ Subsequently, under uniaxial forces, chitosan chains slide and align closely with the aid of reconfigurable SDS micelles. Finally, the aligned micellar Chit‐H^+^/SDS hydrogels are immersed in NaOH solution to remove the sacrificial SDS micelles and enable H‐bond reforming. More crucially, the aligned network is well maintained and stabilized by the reconstructed H‐bond crystalline network.

The resultant anisotropic chitosan hydrogels films are transparent, ultrathin (4–98 µm) yet mechanically strong. Compared to the traditional strategy, the strength increases 140‐fold, from 0.4 ± 0.1 to 58.9 ± 3.4 MPa; the modulus increases 595‐fold, from 0.4 ± 0.1 to 226.4± 42.8 MPa; the toughness increases 103‐fold, from 0.1 ± 0.01 to 10.3 ± 2.4 MJ m^−3^ (Figure 1B). After drying‐rehydration, the strength and modulus further rise to 70.3 ± 2.4 and 403.5 ± 76.3 MPa, representing a milestone in extremely high‐strength pure chitosan hydrogel films. The maximum strength is comparable to that of tendons, and the modulus matches or even surpasses that of connective tissues such as cartilage, ligaments, and tendons. Additionally, we constructed a hierarchical hydrogel with Bouligand architectures by assembling five ultrathin anisotropic chitosan hydrogel films. The designed hydrogel closely resembles the lobster underbelly soft membrane in terms of composition (pure chitosan), structure (Bouligand‐type), properties (mechanical strength: 28.2 ± 2.0 MPa, water content: 70.4 ± 3.0%) and appearance (transparent). More competitively, the replica possesses remarkable impact‐resistant performance (up to 6.8 ± 1.0 kJ m^−1^), outperforming the lobster shell (0.4 kJ m^−1^) and impact‐resistant materials designed via other biomimetic strategies (poly(vinyl alcohol) (PVA)/graphene oxide (GO)PVA/GO hydrogels 2.1 kJ m^−1^,^[^ ^2^ ^]^ Cellulose gel/silk fibers 3.1 kJ m^−1^^[^ ^18^ ^]^), thereby promising the flexible hydrogels as impact‐resistant materials. We anticipate that the sacrificial micelle‐assisted‐alignment strategy could provide new perspectives for the design of high‐performance polymer hydrogels.

## 2 Results and Discussion

### 2.1 Well‐Aligned Networks and Mechanical Properties of Pure Chitosan Hydrogels via the Sacrificial Micelle‐Assisted‐Alignment Strategy

**Figure 2:**
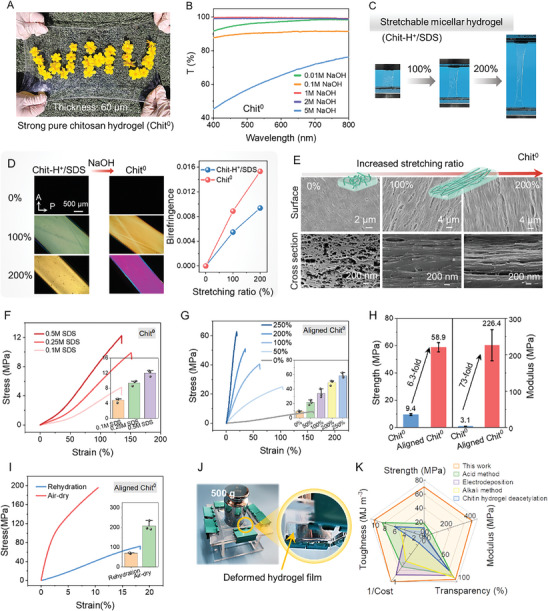
Well‐aligned networks and mechanical properties of pure chitosan hydrogels via the sacrificial micelle‐assisted‐alignment strategy. A) A photograph of the transparent, pure chitosan hydrogel film (Chit^0^) with an area of 15×25 cm^2^ (≈60 µm in thickness). B) Transparency of pure chitosan hydrogel films treated with various NaOH concentrations. C) Photos of Chit‐H^+^/SDS hydrogel films under uniaxial stretching with various stretching ratios. D) POM images and birefringence of Chit‐H^+^/SDS and Chit^0^ with various stretching ratios (P: polarizer, A: analyzer). E) SEM images of Chit^0^ with various stretching ratios. F) Representative tensile curves of Chit^0^ without alignment crosslinked with various SDS concentration. G) Representative tensile curves of Aligned Chit^0^ with various stretching ratios. Inserted chart shows the raw data points and mean ± SD, n = 3. H) Sacrificial micelle‐assisted‐alignment strategy contributes to strength and modulus reinforcement. I) Representative tensile curves of Aligned Chit^0^ after air‐drying and rehydration. J) A 500 g weight is placed on the ultrathin Chit^0^ hydrogel films (≈60 µm in thickness). The film shows deformation but no rupture. K) Comparison of mechanical properties, transparency and cost of pure chitosan hydrogels among the traditional acid method,^[^ ^10^, ^11^, ^12^, ^13^ ^]^ the alkali method,^[^ ^8^, ^10^, ^33^, ^34^ ^]^ electrodeposition,^[^ ^35^, ^36^, ^37^ ^]^ and chitin hydrogel deacetylation.^[^ ^38^ ^]^

Pure chitosan hydrogel films (Chit^0^) were successfully prepared via the sacrificial micelle‐assisted‐alignment strategy (Figure S1, Supporting Information). **Figure** **2A** displays a transparent, pure chitosan hydrogel film with an area of 15 × 25 cm^2^, suggesting the potential for large‐area production. The UV–vis spectra in Figure 2B reveal that the transparency of Chit^0^ hydrogel films varies with the concentration of NaOH. Optimally, the optical transmittance of Chit^0^ hydrogel films regenerated in 1 m NaOH is above 95%. To introduce an aligned network, micellar Chit‐H^+^/SDS hydrogel films are uniaxially stretched previous to NaOH treatment, as shown in Figure 2C. Owing to the viscoelasticity, the micellar Chit‐H^+^/SDS hydrogel film is readily stretchable (ultimate strain up to 280.5 ± 7.3%) (Figure S2, Supporting Information). This deformation can be fully maintained after the removal of external forces. It has been reported that the SDS micelles merge into larger micelles to bear the load in Chit‐H^+^/SDS networks at the nano scale, endowing the viscoelastic hydrogel with outstanding strength.^[^ ^19^ ^]^ The uniaxial forces also endow the micellar Chit‐H^+^/SDS and Chit^0^ hydrogel films with anisotropic structures, as demonstrated by polarizing optical microscopy (Figure 2D; Figure S3, Supporting Information). Under uniaxial stretching, birefringence colors appear, signifying anisotropic structures.^[^ ^6^ ^]^ Notably, with increasing stretching ratios, the color progression is unidirectional as per the Michel‐Levy chart, which allows to corelate the exhibited colors with the numerical birefringence and film thickness.^[^ ^20^, ^21^ ^]^ As the stretching ratio increases, the birefringence rises from 0 to 0.009 for the micellar Chit‐H^+^/SDS hydrogel. After NaOH treatment, the micellar hydrogels transform into pure chitosan hydrogels and the birefringence further increases to 0.015, which surpasses the natural tissue with organized fibrous structure including cat corneas (0.003),^[^ ^22^ ^]^ rat tail tendons (0.003),^[^ ^23^ ^]^ human corneas (0.00159),^[^ ^24^ ^]^ muscles (0.002),^[^ ^25^ ^]^ human tendons (0.012).^[^ ^26^ ^]^ With uniaxial stretching, coiled and randomly‐distributed chitosan nanofibers become extended and well‐aligned, as indicated by SEM and AFM images (Figure 2E; Figure S4, Supporting Information). Meanwhile, nano‐porous networks convert to compact networks.

The mechanical properties of pure chitosan hydrogel films are affected by Chit‐H^+^/SDS reaction time, SDS concentration, NaOH concentration, stretching ratio (Figure 2F–H; Figure S5–S7, Supporting Information). Strikingly, compared to the Chit^0^ hydrogel films (9.4 ± 0.7 MPa), the Aligned Chit^0^ hydrogel films show an ultimate strength of 58.9 ± 3.4 MPa and an over 73‐fold increase in modulus (from 3.1 ± 0.3 to 226.4 ± 42.8 MPa) (Figure 2G,H).The remarkable increment in mechanical strength and modulus can be rationalized that the adjacent chitosan chains well align and closely stack via uniaxial stretching, which favors the crystallite formation via reconfiguration of hydrogen bonds and hydrophobic interactions during NaOH treatment.^[^ ^27^, ^28^, ^29^ ^]^ After air‐drying and rehydration, the strength and modulus dramatically rise to 70.3 ± 2.4 and 403.5 ± 76.3 MPa (Figure 2I). During drying process, the collapse of nanopores allow the self‐densification of chitosan networks.^[^ ^30^ ^]^ The maximum strength ≈70.3 MPa is on par with the tendon (55–120 MPa)^[^ ^31^ ^]^ and the modulus is comparable to or even outperform that of the connective tissue like cartilage, ligament, tendon.^[^ ^32^ ^]^ Besides, water content decreases with increasing SDS concentration and stretching ratio, from 84.3 ± 1.4% to 75.7 ± 2.1%. After air‐drying and rehydration, water content further declines to 64.4 ± 2.2%. Figure 2J displays an ultrathin Chit^0^ hydrogel film supporting a 500 g weight, accompanied with film deformation but no rupture. The radar chart compares the mechanical properties, transparency and cost of regenerated pure chitosan hydrogels among various methods, highlighting the advantages of sacrificial micelle‐assisted‐alignment strategy. (Figure 2K; Table S1, Supporting Information).

### 2.2 The Removal of Micelles and Regeneration of Ultrathin Pure Chitosan Hydrogel Films

**Figure 3:**
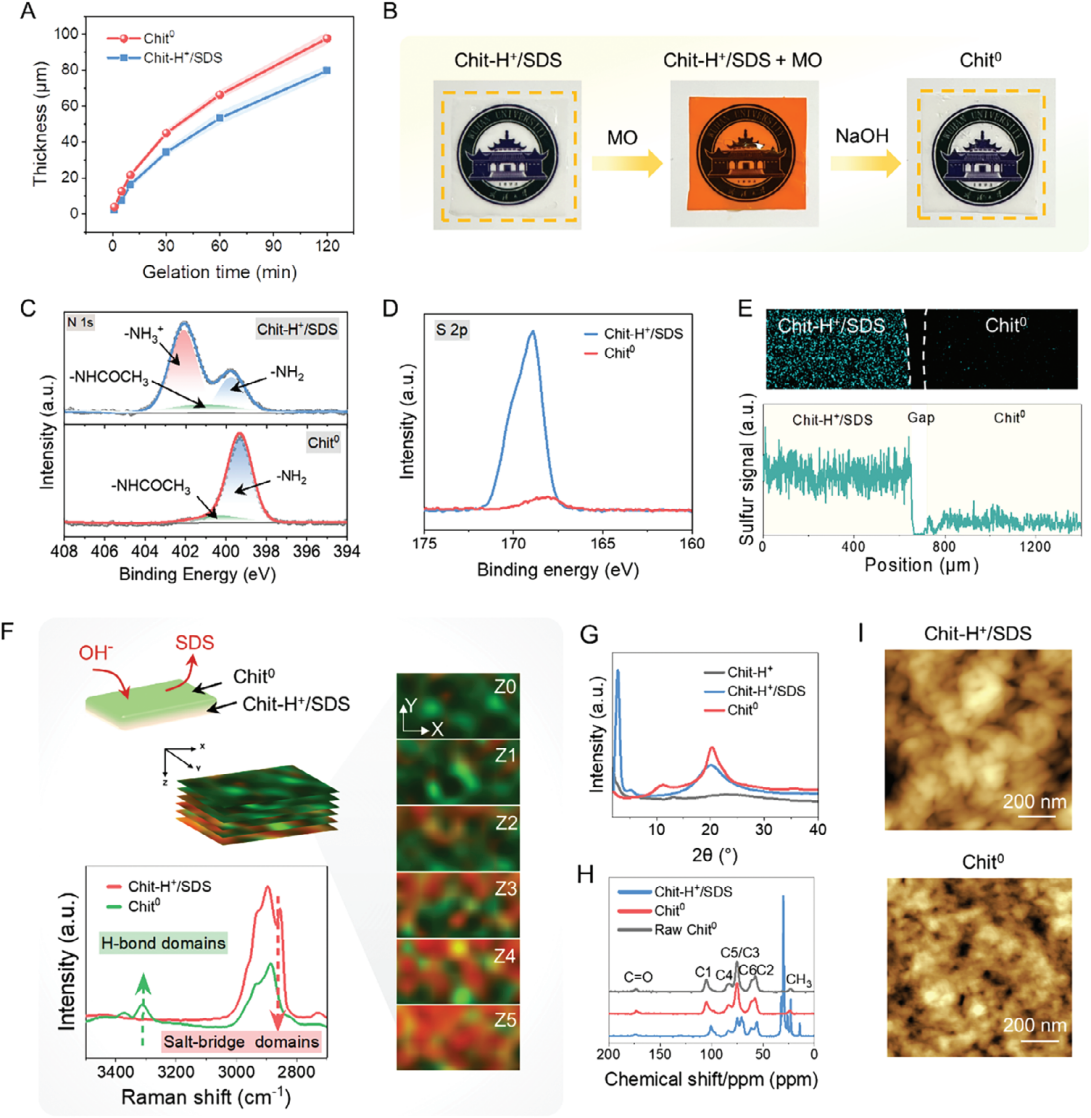
The removal of micelles and regeneration of ultrathin pure chitosan hydrogel films. A) Effects of gelation time on the thickness of Chit‐H^+^/SDS and Chit^0^ hydrogel films. B) The transparent Chit‐H^+^/SDS hydrogels are soaked in 0.1% methyl orange (MO) solution and then treated with 1 m NaOH. The transparent films are highlighted by yellow dashed boxes. XPS high‐resolution scans of C) N_1s_ and D) S_2p_ for Chit‐H^+^/SDS and Chit^0^. E) EDS mapping and line scan suggest the different distribution of sulfur on Chit‐H^+^/SDS and Chit^0^. F) In situ Raman mapping for visualization of structural changes from Chit‐H^+^/SDS to Chit^0^. G) XRD curves of Chit‐H^+^, Chit‐H^+^/SDS and Chit^0^. H) ^13^C CP/MAS NMR spectra of Chit‐H^+^/SDS, regenerated pure chitosan (Chit^0^) and raw chitosan. I) AFM images of Chit‐H^+^/SDS and Chit^0^.

After the alignment process, the SDS micelles, acting as sacrificial templates, can be removed via NaOH treatment, generating anisotropic pure chitosan hydrogel films. The micellar Chit‐H^+^/SDS hydrogel films transform into pure chitosan hydrogel films, accompanied with slight swelling in thickness (Figure S8, Supporting Information). The thickness of chitosan hydrogel films increases with gelation time, while decreases with increased chitosan and SDS concentrations, ranging from 4 ± 1 to 98 ± 4 µm, showing that our method is efficient in fabricating thin films with micrometer in thickness (**Figure** **3A**; Figure S9, Supporting Information). To plainly exhibit the discrepancy between Chit‐H^+^/SDS and Chit^0^ on a macroscopic scale, Chit‐H^+^/SDS hydrogel films are soaked in 0.1% methyl orange (MO) solution and then treated with 1 m NaOH. After soaking in MO solution, the transparent Chit‐H^+^/SDS micellar hydrogel turns orange due to the absorption of MO. Subsequently, the hydrogel film becomes transparent again after NaOH treatment, implying the desorption of MO due to the elimination of SDS (Figure 3B).

The successful removal of SDS was further evidenced by X‐ray photoelectron spectroscopy, energy dispersive spectroscopy and Raman spectroscopy. i) X‐ray photoelectron spectroscopy (Figure 3C,D; Figure S10, Supporting Information); After NaOH treatment, ‐NH_3_^+^ converts to ‐NH_2_ and S_2p_ peak disappears, proving that the protonated chitosan chains become deprotonated and SDS micelles are successfully removed.^[^ ^39^, ^40^ ^]^ ii) Energy dispersive spectroscopy (Figure 3E; Figure S11, Supporting Information); EDS mapping, line scan and spectra suggest that the sulfur evenly distributes on Chit‐H^+^/SDS and is barely detectable on Chit^0^. iii) Raman spectroscopy (Figure 3F; Figure S12, Supporting Information);^[^ ^41^, ^42^ ^]^ In 2D Raman maps, the red region represents the amine‐sulfate salt‐bridge network between chitosan and SDS and the green region denotes the H‐bond crystalline network in chitosan. Chit‐H^+^/SDS polyelectrolytes complexes are mainly dominant by amine‐sulfate salt‐bridges, while in the Chit° crystalline network, H‐bonds reconstructed. Moreover, the dynamic process for transformation of Chit‐H^+^/SDS to Chit^0^ is justified through in situ Raman mapping.^[^ ^43^ ^]^ After submerging Chit‐H^+^/SDS hydrogel films in 0.01 m NaOH, the transition of H‐bond crystalline networks vertically evolves from the surface to the interior (from Z_0_ to Z_5_, step length: 2 µm), which is consistent with the diffusion direction of NaOH. The bands in Chit^0^ at 3309 cm^−1^ is credited to ‐OH stretching vibration, which is absent from Chit‐H^+^/SDS.^[^ ^44^, ^45^, ^46^ ^]^ 1D Raman patterns derived from the Raman mappings validate the same result. XRD measurements also confirm that the Chit^0^ recovers orthorhombic hydrated crystallites (*P*2_1_2_1_2_1_ space group), which are stabilized by reconstructed H‐bonds (Figure 3G).^[^ ^47^, ^48^, ^49^ ^]^ When chitosan is dissolved in hydrochloric acid (Chit‐H^+^), no crystalline peaks appear in the XRD pattern, indicating that the crystalline structure is disrupted, i.e., the H‐ bonds are broken. In Chit‐H^+^/SDS, new characteristic peaks appear, which are completely different from the characteristic peaks of pure chitosan, indicating the formation of a new ordered structure caused by the SDS micelles.^[^ ^17^ ^]^ After NaOH treatment, SDS is removed, resulting in pure chitosan, and the characteristic peaks of the crystalline structure of chitosan reappear, indicating the reconstruction of hydrogen bonds. FTIR also demonstrates the reconstruction of H‐bonds (Figure S13A, Supporting Information). It is noteworthy that the chemical structure and the thermal stability of chitosan are well preserved after NaOH treatment, which is clarified by the cross polarization/magic angle spinning (CP/MAS) ^13^C solid nuclear magnetic measurements and thermogravimetric analyses (Figure 3H; Table S2 and Figure S13B,C, Supporting Information). The degree of deacetylation of raw chitosan and regenerated chitosan calculated from NMR curves are 85.3% and 84.6%, respectively. Nanopores appear in Chit^0^ after NaOH treatment, as proven by AFM images and SEM images (Figure 3I; Figure S14, Supporting Information). These results jointly demonstrate that 1) the regenerated chitosan hydrogel films are ultrathin (4–98 µm); 2) after NaOH treatment, SDS micelles acting as sacrificial templates can be removed, and chitosan chains reassociate into the H‐bond crystalline network; 3) the regeneration process does not compromise the chemical structure and the thermal stability of chitosan.

### 2.3 Mechanisms for Mechanically Strong, Anisotropic Chitosan Hydrogel Films

**Figure 4:**
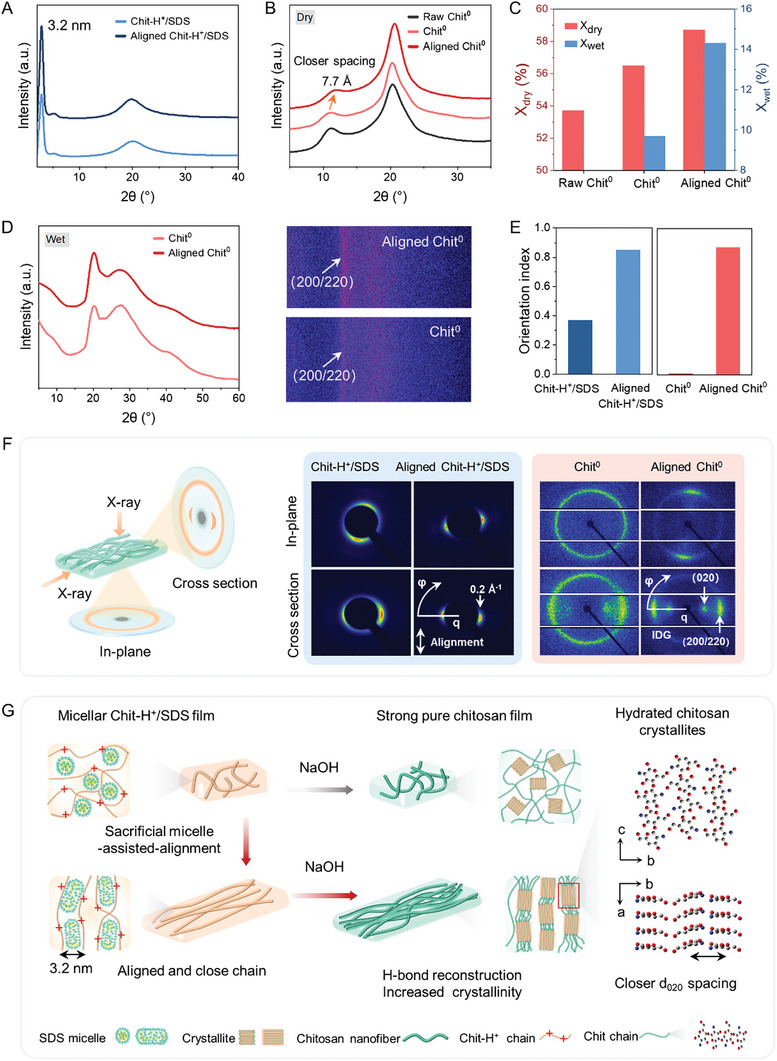
Mechanisms for mechanically strong, aligned chitosan hydrogel films via the sacrificial micelle‐assisted‐alignment strategy. XRD curves of A) Chit‐H^+^/SDS and Aligned Chit‐H^+^/SDS, B) raw chitosan, Chit^0^ and Aligned Chit^0^ in the dry state. Aligned Chit‐H^+^/SDS and Aligned Chit° correspond to a stretching ratio of 200%. C) Crystallinity of raw chitosan and Chit^0^ and Aligned Chit^0^ in the dry/wet state. D) XRD curves and corresponding 2D patterns of Chit^0^ and Aligned Chit^0^ in the wet state. E) Orientation indices (f_c_) and F) 2D WAXS patterns of the Chit‐H^+^/SDS, Aligned Chit‐H^+^/SDS, Chit^0^ and Aligned Chit^0^. The in‐plane denotes that X‐ray beam is perpendicular to the film surface and the cross section denotes that X‐ray beam is parallel to the film surface. *q* is scattering vector and IDG represents the intermodular detector gap. G) Proposed mechanisms for the formation of mechanically strong, anisotropic chitosan films. The grey, blue, red spheres denote carbon, nitrogen and oxygen atoms. Hydrogen atoms and water molecules inside the hydrated chitosan crystallites are not shown for clarity.

The mechanisms for mechanically strong, anisotropic chitosan hydrogel films were clarified by the crystallographic study, stressing the influence of sacrificial micelle‐assisted‐alignment on the nanoscale architecture of chitosan. As shown in **Figure** **4A**, the XRD curve of Chit‐H^+^/SDS displays a sharp diffraction peak at 2.8°, indicating an interplanar spacing of 3.2 nm, which is consistent with the size of SDS micelles that intercalated between chitosan chains.^[^ ^50^ ^]^ The XRD curves of raw chitosan and Chit^0^ indicate a typical hydrated chitosan morphology (Figure 4B), as evidenced by the characteristic peaks at ≈10.2°, 20.3° corresponding to (020), (200)/(220) plane.^[^ ^19^, ^47^ ^]^ Notably, in Aligned Chit^0^, the diffraction peak of (020) shifts toward larger angles, demonstrating a closer interplanar spacing of the (020) plane from 8.1 to 7.7 Å, and the peak of (200)/(220) intensifies, confirming an increased crystallite size from 3.0 to 3.2 nm. In addition, the crystallinity exhibits a 2.2% increase in the dry state (from 56.5% to 58.7%) and a 4.6% increase in the wet state (from 9.7% to 14.3%) (Figure 4C). In the wet state, the broad diffraction peaks at 27.6° and 40.1° stem from the scattering of water in the chitosan hydrogels^[^ ^8^ ^]^ and the characteristic peak of (200)/(220) shifts toward smaller diffraction angles, revealing an increased interplanar spacing in chitosan crystallites (Figure 4D).

2D WAXS confirmed the oriented structure caused by sacrificial micelle‐assisted‐alignment, as shown Figure 4E,F. In Chit‐H^+^/SDS, the equatorial arc pattern narrows into a spot after alignment, indicating an increased anisotropy. In Chit° films, isotropic ring patterns are observed, symbolizing the random distribution of chitosan chains in the plane. Upon stretching, the equatorial arc patterns appear in Aligned Chit° films since the chitosan crystallites align in the stretching direction, which also represents the nanofibrils orientation direction.^[^ ^51^ ^]^ Furthermore, to quantitatively compare the alignment of the chitosan chains in Chit‐H^+^/SDS, Aligned Chit‐H^+^/SDS, Chit^0^ and Aligned Chit^0^, orientation indices (f_c_) are calculated from azimuthal integration profiles (Figure S15, Supporting Information).^[^ ^52^ ^]^ The orientation index is defined on a scale from 0 to 1, where 0 refers to random distribution and 1 corresponds to perfect alignment. The orientation indices for Chit‐H^+^/SDS, Chit^0^, Aligned Chit‐H^+^/SDS, and Aligned Chit^0^ are 0.37, 0.01, 0.85, and 0.87, respectively. The sacrificial micelles assist the alignment of chitosan chains, and this alignment is effectively preserved after alkali treatment.

Figure 4G is a schematic illustration for the formation of mechanically strong, anisotropic chitosan films via sacrificial micelle‐assisted‐alignment strategy. In micellar Chit‐H^+^/SDS hydrogels, reconfigurable SDS micelles change from spherical to cylindrical shapes under uniaxial forces,^[^ ^19^ ^]^ which effectively guide the sliding and alignment of protonated chitosan chains and avoid fracture. This process allows the protonated chitosan chains with more extended conformation, closer interchain spacing (3.2 nm). After NaOH treatment, SDS micelles, acting as sacrificial templates, can be removed. Simultaneously, with the closer interchain spacing, adjacent chitosan chains tightly pack via H‐bond reconfiguration, generating compact hydrated chitosan crystallites with closer lattice plane spacing (*d*_020_,7.7 Å) and larger dimensions. This process enhances the crystallinity of the chitosan films, thereby improving the mechanical performance. Regarding the nano architectures of hydrated chitosan crystallites, the chitosan chains are twofold helix along the *c* direction (the direction of the molecular chains) and antiparallel‐packed along the *b* direction, generating a sheet structure parallel to the *bc*‐plane. These sheets are further packed along *a* axis direction, forming an orthorhombic hydrated crystallites (*P*2_1_2_1_2_1_ space group). The adjacent sheets are stabilized by the water‐bridges between chitosan chains.^[^ ^47^, ^48^, ^49^ ^]^

### 2.4 Design and Characterization of the Lobster Underbelly Soft Membrane Replica

**Figure 5:**
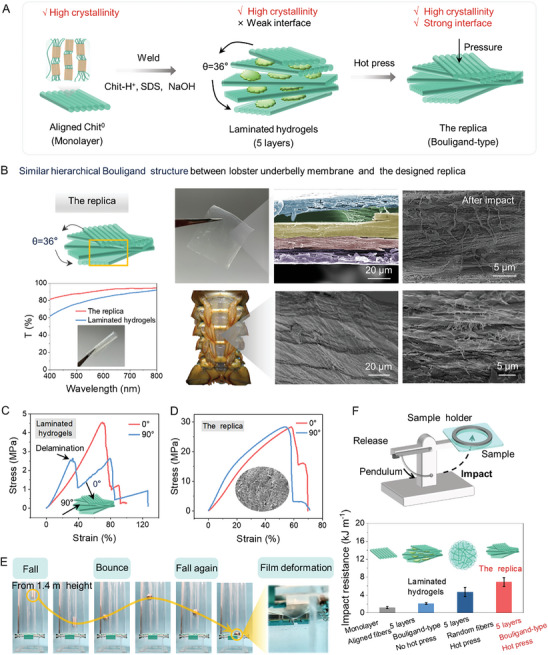
Design and characterization of the lobster underbelly soft membrane replica. A) Schematic of the biomimetic assemble strategy for the replica. B) Similar hierarchical Bouligand structure between lobster underbelly membrane and the designed replica. Transparency of laminated hydrogels and the replica. Inset is a photo of the transparent and pliable replica with a size of 4 × 4 cm^2^. Representative tensile curves of C) laminated hydrogels and D) the replica with the loading direction of θ = 0° and θ = 90°. θ is defined as the angle between the tensile loading and the direction of aligned nanofibers in the first layer. The inset is the SEM image of the replica after tensile test, showing fibrous morphology. E) Metal ball impact experiments of the replica. F) Schematic set‐up of the impact resistant test and excellent impact resistance of the replica.

Recent advances have revealed that the lobster underbelly soft membrane is a transparent natural hydrogel with exceptional strength (up to 23.4 MPa), despite high water content (75–90%).^[^ ^53^ ^]^ In the fibrous multi‐layer Bouligand structure, the chitin nanofibers in each layer rotates by 36° relative to the adjacent one, which plays a crucial role in dissipating energy during rupture. However, the precise replication of such delicate structures remains challenging. Given the high crystallinity of the strong aligned chitosan hydrogel films, we anticipate that they could function as robust building blocks to replicate the soft yet mechanically strong lobster underbelly soft membrane, as shown in **Figure** **5A**. Specifically, the elementary building block is the Aligned Chit^0^ hydrogel film (monolayer), which is composed of aligned chitosan nanofibers and possesses high crystallinity (Figure S16, Supporting Information). Five layers Aligned Chit^0^ hydrogel films are assembled into the Bouligand structure, in which the adjacent layer rotates with an angle of ≈36°. The layers are welded into laminated hydrogels by applying concentrated Chit‐H^+^ solutions between layers, soaking in SDS solution and then alkali solution. Ultimately, hot press is enlisted as an effective strategy to introduce strong interfaces between layers in the replica. The Bouligand structure and the strong interfaces between fibrous layers closely resemble the architectures of the lobster underbelly soft membrane (Figure 5B; Figure S17, Supporting Information). Water content is 82.6 ± 3.5% for laminated hydrogels and 70.4 ± 3.0% for the replica. In the SEM image of the replica, the respective layers are identified with different pseudo‐colors. When it comes to mechanical performance, monolayer hydrogels are anisotropic (Figure S18, Supporting Information), while the laminated hydrogels and the replica are isotropic in‐plane owing to the Bouligand structure (Figure 5C,D). The inflection points in tensile curves of laminated hydrogels verify the delamination of layers during tensile loading, which is due to the weak interfaces between layers. Compared to the laminated hydrogels (4.8 ± 0.9 MPa in the loading direction of 0° and 3.2 ± 0.5 MPa in the loading direction of 90°), the tensile strength of the replica can reach as high as 28.2 ± 2.0 MPa in the loading direction of 90° (29.8 ± 1.3 MPa in the loading direction of 0°) because of the strong interfaces. The loading stress efficiently distributes by chains sliding, fiber pull out, crack twisting among the aligned lamellar Bouligand structure and strong interfaces (Figure S19, Supporting Information).^[^ ^3^, ^54^, ^55^ ^]^ A metal ball (diameter: 25 mm, mass: 65 g) falls on the replica from 1.4 m height and bounces, while the replica shows slight film deformation with no breakage (Figure 5E; Figure S20, Supporting Information).

To quantify the impact resistance of the replica, impact resistance tests were conducted according to ASTM D3420. The set‐up is shown in Figure 5H. The impact resistance of the replica reaches 6.8 ± 1.0 kJ m^−1^, which is superior to that of the monolayer (1.1 ± 0.2 kJ m^−1^) and laminated hydrogels (2.1 ± 0.2 kJ m^−1^) (Figure 5I). Strikingly, the impact resistance of the replica even exceeds the lobster shell (0.4 kJ m^−1^) and other impact‐resistant materials manufactured via other biomimetic strategies (PVA/GO hydrogels 2.1 kJ m^−1^,^[^ ^2^ ^]^ Cellulose gel/silk fibers 3.1 kJ m^−1^^[^ ^18^ ^]^) (Figure S21 and Table S3, Supporting Information). The above results confirm the exquisite replica of the lobster underbelly soft membrane and the potential applications of the designed hydrogels as flexible yet impact‐resistant materials.

## 3 Conclusion

In summary, we proposed a sacrificial micelle‐assisted‐alignment strategy to significantly enhance the mechanical properties of polymer hydrogels by adjusting the sequence of chain alignment and H‐bond formation. In this method, SDS micelles guide the sliding and alignment of semi‐flexible chitosan chains through dynamic morphological reconfiguration and electrostatic attractions under uniaxial forces. Subsequently, SDS micelles, acting as sacrificial templates, are completely removed via NaOH treatment, lead to the reforming of H‐bond chitosan crystalline networks. The resultant pure chitosan hydrogel films are ultrathin with a thickness of 4 ± 1 to 98 ± 4 µm. Compared to the traditional method, the strength, modulus, and toughness have increased by 140‐fold (0.4 ± 0.1 to 58.9 ± 3.4 MPa), 595‐fold (0.4 ± 0.1 to 226.4± 42.8 MPa), and 103‐fold (0.1 ± 0.01 to 10.3 ± 2.4 MJ m^−3^), respectively, which remarkably outperforms the reported pure chitosan hydrogels developed by other strategies. It is proved that the sacrificial micelle‐assisted‐alignment strategy endows the regenerated pure chitosan with closer interchain spacing, larger hydrated chitosan nano crystallites, well‐aligned chitosan chains and enhanced crystallinity, which are conducive to energy dissipation and reinforced mechanical performance. Besides, the strong pure chitosan hydrogels are assembled into hierarchical hydrogels with Bouligand structure and strong interfaces between adjacent monolayers. The biomimetic hydrogels are similar to the soft membrane of the lobster underbelly, in terms of composition (pure chitosan), structure (Bouligand‐type), properties (mechanical strength: 28.2 ± 2.0 MPa, water content: 70.4 ± 3.0%), appearance (transparent). More attractively, the flexible replica exhibits favorable impact resistance (up to 6.8 ± 1.0 kJ m^−1^). This work offers novel insights for the design of high‐performance polymer hydrogels and demonstrates the biomimetic materials with impact resistance through optimizing hierarchical structures, rather than introducing hazardous cross‐linkers or other components.

## 4 Experimental Section

### Materials

Chitosan (Chit, *M_w_*: 4.06×10^5 ^g mol^−1^, deacetylation degree: 85%) was supplied by Tokyo Chemical Industry Co., Ltd (Japan). Hydrochloric acid (HCl), sodium dodecyl sulfate (SDS), sodium hydroxide (NaOH) were obtained from Sinopharm Chemical Reagent Co., Ltd. (China). All reagents were used without further purification.

### Preparation of Regenerated Chitosan Hydrogel Films (Chit^0^ and Aligned Chit^0^)

1 wt.% chitosan solution was prepared by dissolving a certain amount of chitosan flakes into diluted HCl solution and mechanically stirred for 12 h at ambient temperature. The pH is controlled at 5–6. SDS solution was prepared by dissolving a certain amount of SDS powder into deionized water and then subjected to ultrasound for 5 min to eliminate the bubbles. In a typical procedure, 1 wt.% chitosan solution was cast in the Petri dish and then exposed to a bulk SDS solution (0.25 m), during which the gelation interfaces advanced vertically (from the interfaces of chitosan and SDS solution to the bottom of the petri dish), causing the formation of micellar hydrogels (designated Chit‐H^+^/SDS). Unless otherwise specified, this process lasted for 1 h, after which the petri dish was taken out from the bulk SDS solution. Chit‐H^+^/SDS hydrogel films on the surface were separated from the chitosan solution and rinsed with deionized water. Chit‐H^+^/SDS hydrogel films further underwent uniaxial stretching (designated Aligned Chit‐H^+^/SDS), followed by immersing in NaOH solution for 0.5–24 h depending on the alkali concentration. Ultimately, the as‐prepared hydrogel films were rinsed in abundant water to remove the residual NaOH and SDS, resulting in pure regenerated chitosan hydrogel films (designated Aligned Chit^0^). The pure regenerated chitosan hydrogel films without uniaxial stretching were designated as Chit^0^_._ For comparison, 2.5 wt.% chitosan solution was cast in a petri dish (diameter: 90 mm) and immerged in 1 m NaOH until full gelation.

### Preparation of the Lobster Underbelly Soft Membrane Replica

The monolayer (Aligned Chit^0^) was prepared in the condition of 2 h gelation time, 50% stretching ratio, 1 m alkali regeneration. Five layers of monolayer was assembled into a Bouligand‐type architecture in which each layer rotated by 36° relative to the adjacent one. The adjacent layers were welded into laminated hydrogels through 3 steps. First, 3 wt.% chitosan solution (Chit‐H^+^) was evenly applied between layers. Second, the Bouligand‐type hydrogels were soaked in SDS solution to strengthen the interfaces between layers. Ultimately, the SDS was eliminated through alkali treatment. To obtain the lobster underbelly soft membrane replica, the laminated hydrogels were subjected to hot press (6–8 MPa, 80 °C, 12 h) to introduce strong interfaces between layers.

### Mechanical Tests

The mechanical properties of the hydrogel films were determined by tensile tests on a universal electronic testing machine (CMT6350, Shenzhen SANS Test Machine, China). Specimens (40 mm × 4 mm) were clamped and stretched at a constant speed of 10 mm min^−1^ under uniaxial tension until failure. Stress was defined as the force divided by the initial area of the sample cross‐section. Toughness was the ability of a material to absorb energy and plastically deform without fracturing. Toughness was calculated by integrating the area under the stress–strain curve. Elastic moduli were obtained from the slope of the stress–strain curves in the initial linear region (strain less than 5% for hydrogel films and 1% for dry films). Tensile tests were performed at least three times (n ≥ 3) for each sample, and results were presented as mean value ± standard deviation.

### Impact Resistance Tests

Impact resistance tests were performed on a film impact tester (GBG‐L2, China) based on ASTM D3420. The thickness of samples was determined by thickness gauges. Square samples with sides of 13 cm were fixed by the sample holder (10 cm in diameter). The instrument used a semi‐spherical pendulum to impact and break through the specimen at a certain impact speed, so as to evaluate energy consumed by the pendulum. Impact resistance of the sample was determined by dividing the consumed energy by the thickness. Results were presented as mean value ± standard deviation, n = 3.

### Characterizations

The transparency of the hydrogel films was determined by UV–vis spectrophotometer (Shimadzu UV‐2700, Japan).

The morphologies of the hydrogel films were characterized by field emission scanning electron microscope (FE‐SEM, ZEISS, Germany) and atomic force microscope (AFM, SPM‐9700HT, Japan). Samples for AFM were dried in air. Samples for SEM were frozen in liquid nitrogen, snapped to expose the cross section, freeze‐dried and coated with gold before observation.

X‐ray photoelectron spectroscopy (XPS) data were collected by ESCALAB 250Xi (Thermo Fisher, USA) at constant analyzer energy (CAE) mode. Survey scans were recorded at a pass energy of 100 eV and a step size of 1 eV. High‐resolution scans were collected at a pass energy of 30 eV and a step size of 0.1 eV via 6–18 scans (averaged) in the respective binding energy ranges for N_1s_ and S_2p_. The peaks were calibrated and fitted by software.

The distribution of sulfur on Chit‐H^+^/ SDS and Chit° films was tested on tungsten hairpin filament scanning electron microscope (VEGA Compact, TESCAN, Czech) equipped with energy dispersive spectrometer (Bruker XFlash 6–30) both in the mapping mode and the line scan mode.

Thermogravimetric curves of raw chitosan and pure regenerated chitosan films were obtained by Thermogravimetric Analyzer (DTG‐60, Shimadzu, Japan). The samples were heated from 40 to 600 °C in N_2_ atmosphere.

^13^C CP/MAS Solid State nuclear magnetic resonance (NMR) experiments were conducted on AVANCE NEO 400 MHz NMR spectrometer (Bruker, USA) with a rotation rate of 5000 Hz at 298 K. The acquisition mode is digital quadrature detection and 20 000 scans were performed. The degree of deacetylation (*DD*) were calculated by^[^ ^56^ ^]^

(1) $$DD\;\left(\%\right)=\left[1-\frac{I_{N-CH_{3}}}{\frac{1}{6}\times \left(I_{C_{1}}+I_{I_{C_{2}}}+I_{I_{C_{3}}}+I_{I_{C_{4}}}+I_{I_{C_{5}}}+I_{I_{C_{6}}}\right)}\right]\;\times 100\%$$

where *DD* denotes the degree of deacetylation, *I* denote the integrals of corresponding carbon atom.

Raman spectra and Raman maps were recorded on a confocal Raman microscope (XploRA plus, HORIBA Jobin Yvon, French). The wavelength of the excitation laser is 532 nm with a maximum power of 100 mW. The spatial resolution is 0.5 µm in XY plane and less than 2 µm in Z axial. A Raman map covering an aera of 100×150 µm^2^ was acquired at an acquisition time of 0.1 s. The obtained spectra were calibrated by cosmic ray removal and data smoothing via the accessory software.

Polarized images of the hydrogel films were captured on a polarizing microscope (Leica DM750p, Germany) equipped with a charge coupled device (CCD) camera (Leica ICC50 W, Germany) and a quarter wave plate. The polarizer and the analyzer are at right angle. The spectra of the hydrogel films lay between the polarizer and the analyzer were recorded by fiber optical spectrometer (MAYA2000pro, Ocean Optics, China) with a SMA905 interfaces type collimating eyepiece. Michel‐Levy chart was enlisted to analyze the birefringence that can be determined by dividing the retardation value by the film thickness (d).^[^ ^6^, ^20^, ^21^ ^]^

(2) $$\mathrm{Birefringence}\;=\frac{\mathrm{Retardation}}{d}$$

Crystallographic structures were analyzed by XRD diffractometer (Smartlab SE, Rigaku, Japan) under Cu Kα radiation at 40 kV and 40 mA. All the dry samples were ground into fine particles before tests. For wet samples, hydrogel films were cut into a suitable shape, lying flat on the substrates.

The water content ($f_{H_{2}O}$) was measured by weighing samples before and after drying. Three specimens were used for each test. The crystallinities in the dry state (*X*_dry_) and wet state (X_wet_) were calculated by^[^ ^3^, ^57^ ^]^

(3) $$X_{\mathrm{dry}}=\frac{I_{\mathrm{crystalline}\;}}{I_{\mathrm{crystalline}}+\;I_{\mathrm{amorphous}}}\;$$

(4) $$X_{\mathrm{wet}}=X_{\mathrm{dry}}\;\left(1-f_{H_{2}O}\right)$$

(5) $$f_{H_{2}O}=\frac{m_{\mathrm{wet}}-m_{\mathrm{dry}}}{m_{\mathrm{wet}}}\;$$

where *I*_crystalline _ and *I*_amorphous _denote the crystalline area and amorphous area in the XRD spectra. *m*_wet_ and *m*_dry_ are the mass of the hydrogels before and after drying.

The crystalline size (*D*_hkl_) was calculated based on the Scherrer equation and the interplanar spacing (*d_hkl_*) was determined by Bragg's Law.^[^ ^2^ ^]^

(6) $$D_{\mathrm{hkl}}=\frac{K\lambda }{\beta \cos\theta }\;$$

(7) $$d_{\mathrm{hkl}}=\frac{\lambda }{2\sin\theta }\;$$

where *D*_hkl_ represents the crystallite size perpendicular to the lattice planes. hkl denotes the Miller indices of the selected planes. K is a dimensionless factor related to crystallite shapes. λ is the wavelength of the incident rays. β is the full width at half maximum (FWHM) of the diffraction peak in radians. θ is the diffraction angle.

2D WAXS patterns were obtained on a Small Angle X‐ray scatterometer (Xeuss 2.0, Xenocs, French) equipped with a 2D detector and Geni X 3D light sources (λ = 1.54 Å). Radiation lights are parallel or perpendicular to the films surface. The azimuthal integration profiles of Chit‐H^+^/SDS, Aligned Chit‐H^+^/SDS, Chit^0^ and Aligned Chit^0^ are obtained through the diffraction intensity along the equatorial direction at q = 0.2 Å and q = 1.4 Å ((020)/(200) reticular plane of hydrated chitosan in 2D WAXS patterns). The azimuthal intensity distributions (φ‐I curve) were enlisted to obtain the orientation index (*f_c_*). φ is the azimuthal angle. FWHM is the full width at half maximum of the peak.^[^ ^52^ ^]^

(8) $$f_{c}=\frac{180^{\circ }-\mathrm{FWHM}}{180^{\circ }}\;$$

### Statistical Analysis

For the thickness, mechanical properties (ultimate strain, strength, modulus, toughness), water content, impact resistance, data were presented as mean ± standard deviation (sample size n = 3 for each statistical analysis). Statistical analysis was carried out using Origin software.

## Conflict of Interest

The authors declare no conflict of interest.

## Author Contributions

X.S. and J.Z. conceived the project and wrote the manuscript. X.S., H.D., and Y.D. supervised the work. J.Z. contributed to sample preparation, experiments, and data analysis. Z.Z. and M.W. carried out the metal ball falling test, POM experiments and assisted the photo taking. All authors have commented on and agreed to the published version of the manuscript.

## Supporting information



Supporting Information

## Data Availability

The data that support the findings of this study are available from the corresponding author upon reasonable request.
