# Supplementary material for: Hydrogel Films with Impact Resistance by Sacrificial Micelle‐Assisted‐Alignment
Source: Adv Sci (Weinh). 2024 Oct 7;11(44):2409287. doi: 10.1002/advs.202409287 (PMC11600213; doi:10.1002/advs.202409287)
Supplement: Supplementary file 1 — Supporting Information [file ADVS-11-2409287-s001.docx]

**Supporting Information**

**Hydrogel Films with Impact Resistance by Sacrificial Micelle-Assisted-Alignment**

Jingxian Zhang, Xiaowen Shi*, Zhongtao Zhao, Manya Wang, Hongbing Deng, Yumin Du

*Xiaowen Shi

**Email:**  shixw@whu.edu.cn

**This PDF file includes:**

Figure S1 to S21

Table S1 to S3

SI References

Supporting Information Text


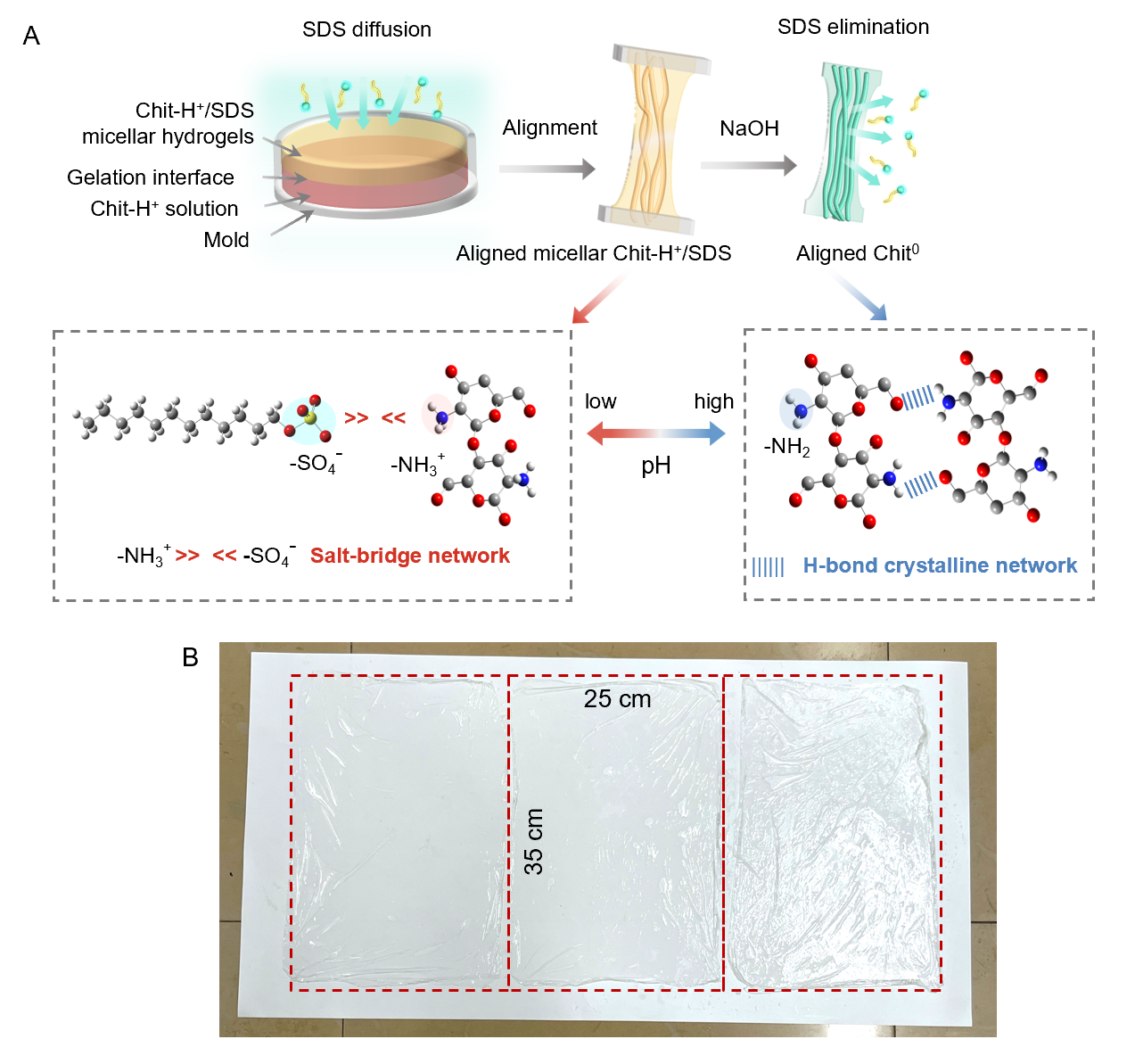


**Figure S1.** (A) Fabrication of strong, pure chitosan hydrogels via sacrificial micelle-assisted-alignment strategy. (B) 3 pieces of hydrogel films with an area of 25×35 cm^2^.

First, chitosan solution was cast in the mold and then exposed to a bulk SDS solution to obtain micellar Chit-H^+^/SDS hydrogels. The strong electrostatic attractions between the positively charged protonated chitosan chains and the negatively charged dodecyl sulfate anion, ion-dipole interactions, hydrophobic interactions and hydrogen bonds synergistically allow the formation of Chit-H^+^/SDS hydrogels via interfacial diffusion induced phase separation.^[1-2]^ Subsequently, the resultant micellar Chit-H^+^/SDS hydrogels were uniaxially stretched and then submerged in NaOH solution to remove the micelles, leading to the strong pure chitosan hydrogel.

The strong electrostatic attractions between the protonated chitosan chains (Chit-H^+^) and dodecyl sulfate ions (DS^-^) generates Chit-H^+^/SDS hydrogel films that are stable even under strong acidic condition. However, with increasing pH, the deprotonated chitosan chains associate into a crystalline network via H-bonds reconstruction, and the SDS are easily removed from the network. Hence, the amine-sulfate salt-bridge network is substituted by the H-bond crystalline network, namely the successful regeneration of pure chitosan. Chemical structures of dodecyl sulfate(C_12_H_25_-SO_4_^-^), protonated chitosan (Chit-NH_3_^+^) and deprotonated chitosan (Chit-NH_2_) are shown. The grey, blue, red white, yellow spheres denote carbon, nitrogen, oxygen, hydrogen and sulfur atoms. In chitosan, only the hydrogen atoms on -NH_3_^+^ and -NH_2_ and are shown for clarity.


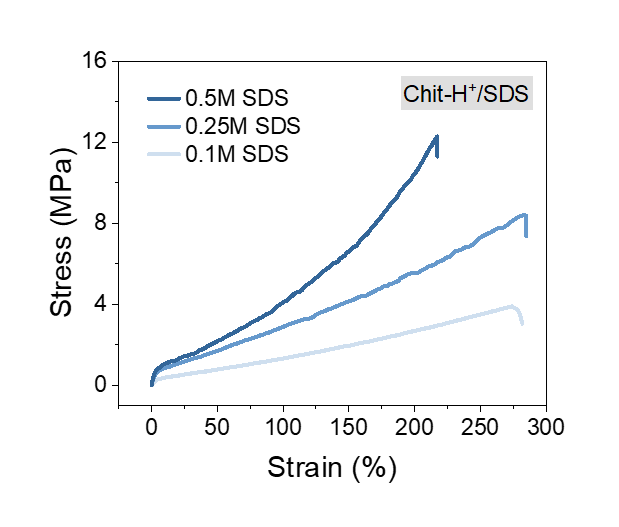


**Figure S2.** Representative tensile curves of micellar Chit-H^+^/SDS without alignment crosslinked with various SDS concentration.


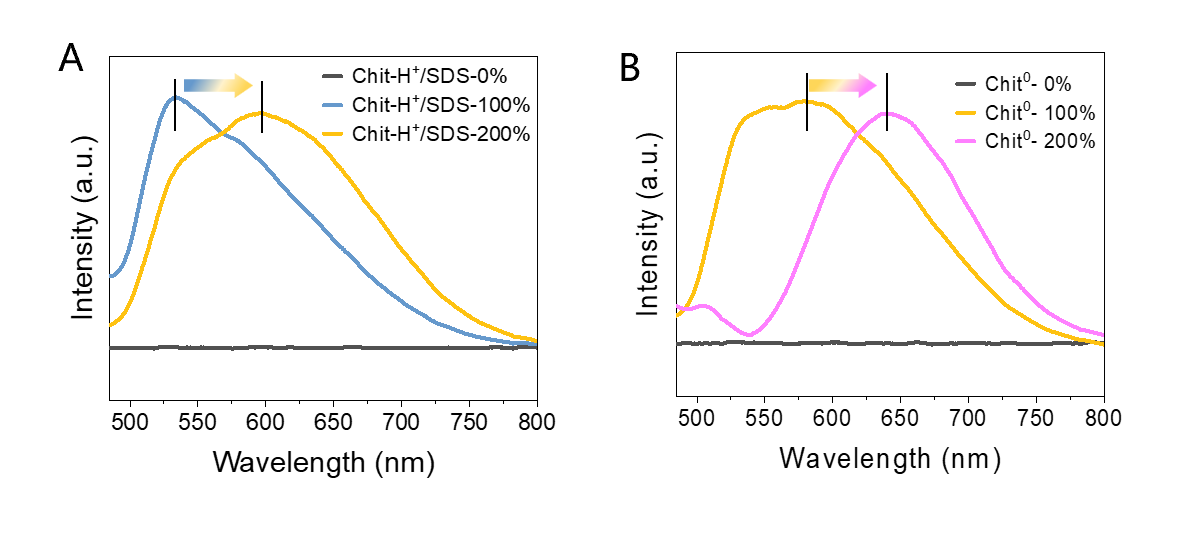


**Figure S3.** (**A** and **B**) Spectra of Chit-H^+^/SDS and Chit^0^ hydrogel films with various stretching ratios.

As the stretching ratio develops from 100% to 200%, the birefringence colors appear and progress from pale blue to yellow in the Chit-H^+^/SDS and from yellow to magenta in the Chit^0^, signifying anisotropic structures. The principal wavelength of the exhibited colors that are recorded by the optical fiber spectrometer.


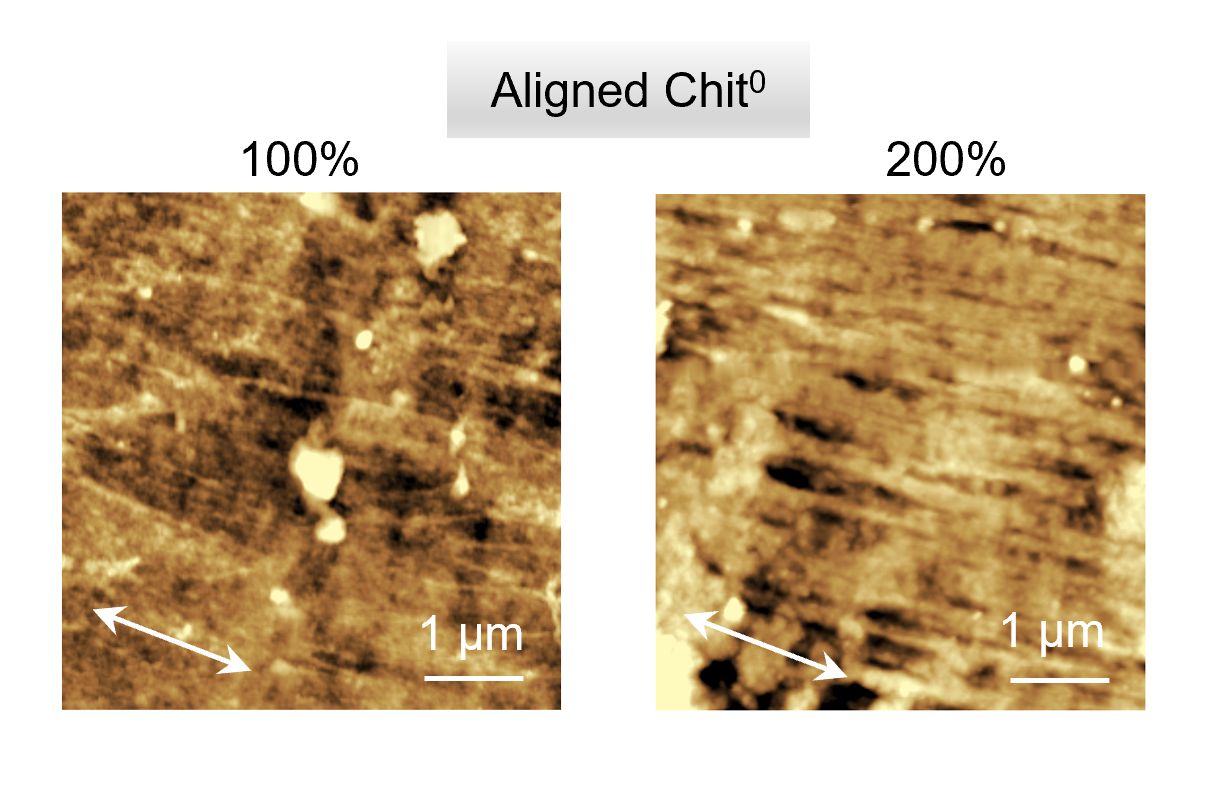


**Figure S4.** AFM images of Chit^0^ with various stretching ratios.


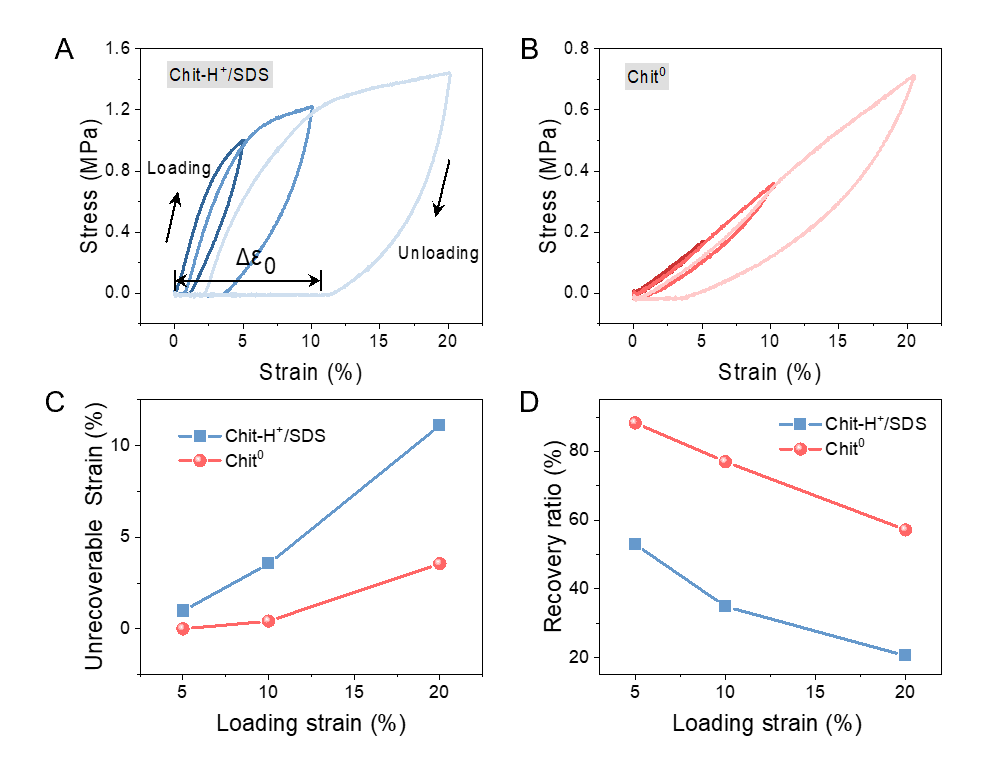


**Figure S5.** (**A** and **B**) Cyclic tensile tests of Chit-H^+^/SDS and Chit^0^. The loading strain increases from 0% ,10% to 20%. (**C** and **D**) Uncoverable strain and recovery ratio of Chit-H^+^/SDS and Chit^0^.

The Chit-H^+^/SDS hydrogel is viscoelastic, while the Chit^0^ hydrogel is elastic. Unrecoverable strain Δε_0_, defined as the difference strains between the loading and unloading curves.^[3]^ Under 5% loading strain, complete shape recovery with Δε_0_ = 0% could be observed for Chit^0^ hydrogel films, while Chit-H^+^/SDS hydrogel films show 1.0% unrecoverable strain. Unrecoverable strain of the Chit-H^+^/SDS hydrogel film further increases to 11.1% under 20% preloading strain. The recovery ratio is defined as the ratio of the second hysteresis area to the first hysteresis area. The recovery performance of Chit^0^ hydrogel films is better than that of the Chit-H^+^/SDS hydrogel films.


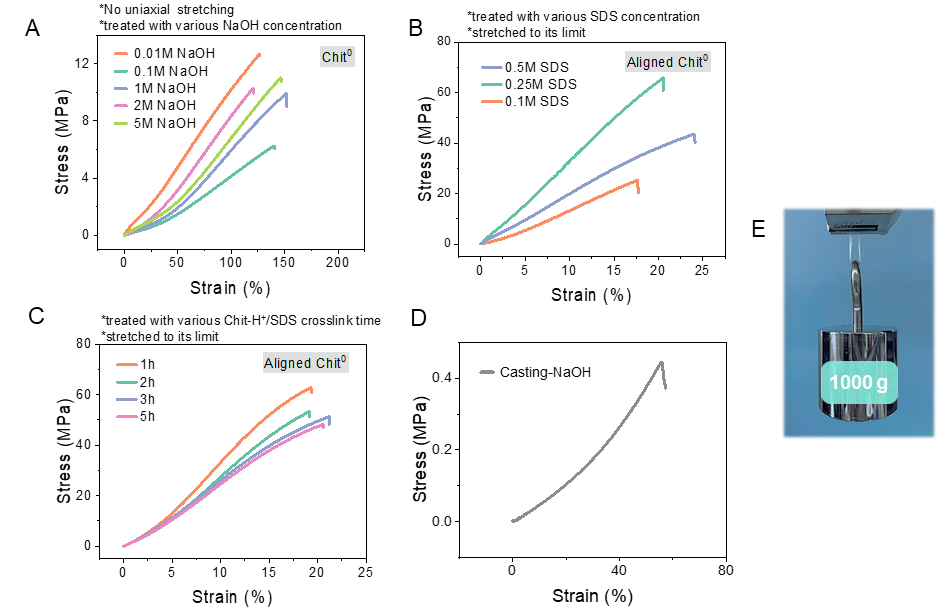


**Figure S6.** Mechanical performance of the regenerated pure chitosan hydrogel films**.** (**A**) Representative tensile curves of Chit^0^ regenerated in alkali with various concentration. (**B** and **C**) Representative tensile curves of Aligned Chit^0^ prepared by various SDS concentration and gelation time. The Chit-H^+^/SDS hydrogels are prepared by various SDS concentration and crosslink time, then stretched to their stretching limit and regenerated by 1 M NaOH. (**D**) Representative tensile curves of the control group. For comparison, 2.5 wt% chitosan solution was cast in a petri dish (diameter: 90 mm) and immerged in 1 M NaOH until full gelation. (**E**) Mechanically strong Aligned Chit^0^ hydrogel film was lifting up the weights of 1000 g.


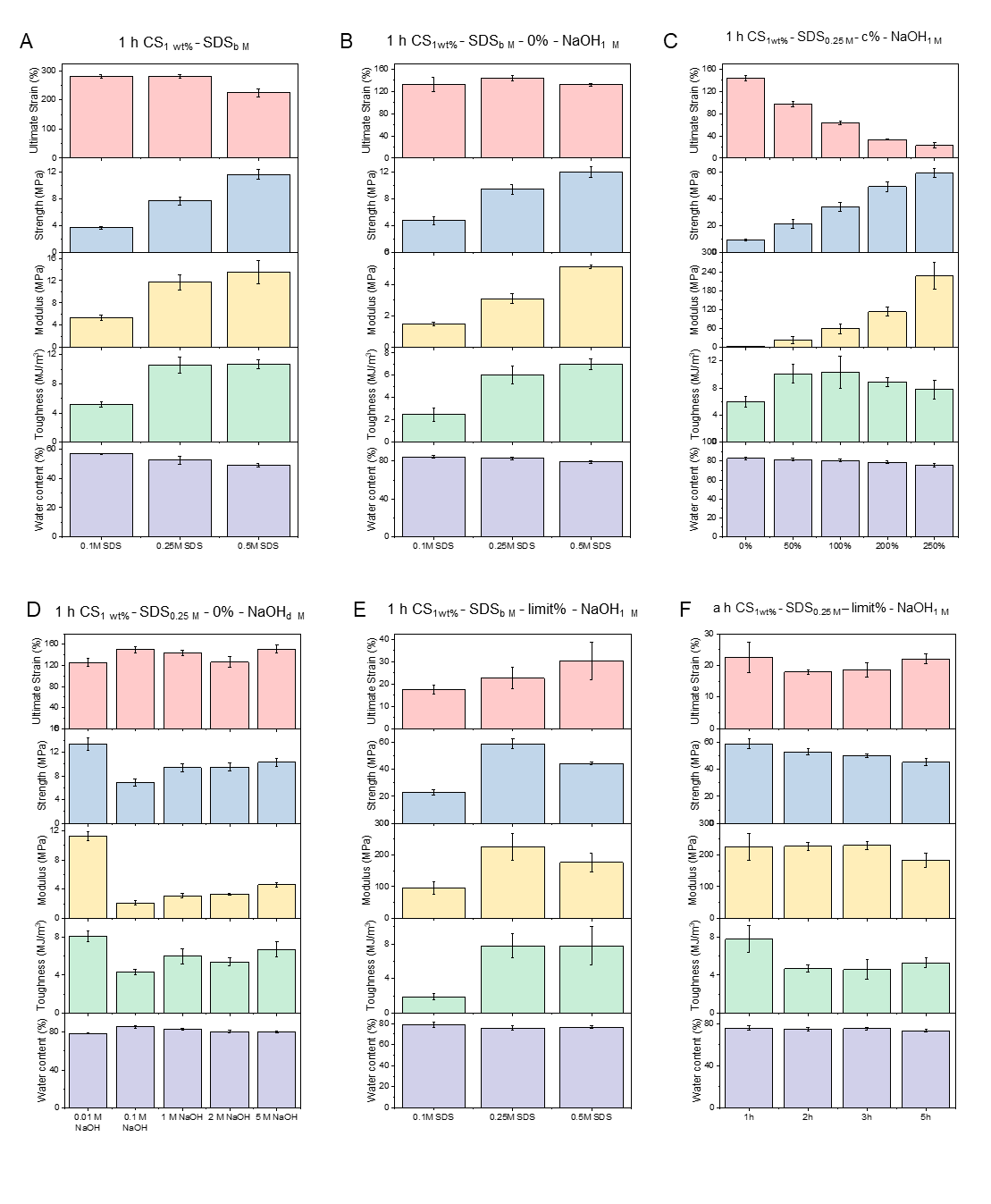


**Figure S7.** Mechanical properties and water content of (**A**) Chit-H^+^/SDS hydrogel films prepared with different SDS; (**B**) the corresponding Chit^0^ hydrogel films; (**C**) the Aligned Chit^0^ hydrogel films. (**D)** the Chit^0^ hydrogel films regenerated in various alkali concentration; (**E**) Aligned Chit^0^ prepared by various SDS concentration; (**F**) Aligned Chit^0^ prepared by various crosslink time. Note: *a, b, c, d* refer to different Chit-H^+^/SDS crosslink time, SDS concentration, stretching ratio, NaOH concentration. Limit% is the stretching limit of corresponding Chit-H^+^/SDS hydrogel film. Unless otherwise noted, the Chit-H^+^/SDS crosslink time is 1h; the SDS concentration is 0.25M; the NaOH concentration is 1M.

For the ultrathin regenerated chitosan hydrogel films, the strength ranges from 4.8 ± 0.6 to 70.3 ± 2.4 MPa; the modulus ranges from 1.5 ± 0.1 to 403.5 ± 76.3 MPa; the toughness ranges from 2.5 ± 0.6 to 10.3 ± 2.4 MJ m^-3^; the ultimate strain ranges from16.8 to 150.9%; the water content ranges from 64.4 ±2.2 to 85.6 ± 1.4%.


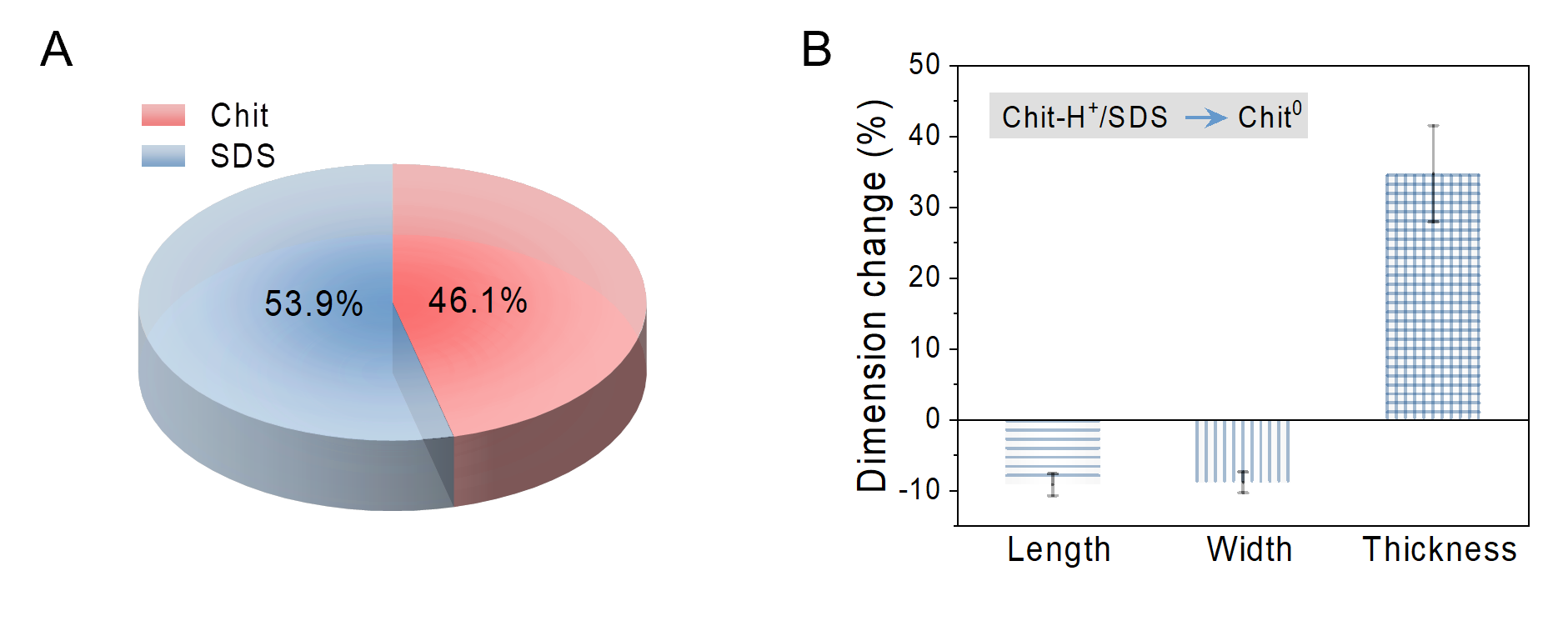


**Figure S8.** (**A**) Composition of the Chit-H^+^/SDS; (**B**) Dimension change of the hydrogels from Chit-H^+^/SDS to Chit^0^_._

The Chit-H^+^/SDS dry films are composed of 53.9% SDS and 46.1% chitosan. During the regeneration process (from Chit-H^+^/SDS to Chit^0^), the hydrogel films show expansion in thickness but contraction in length and width.


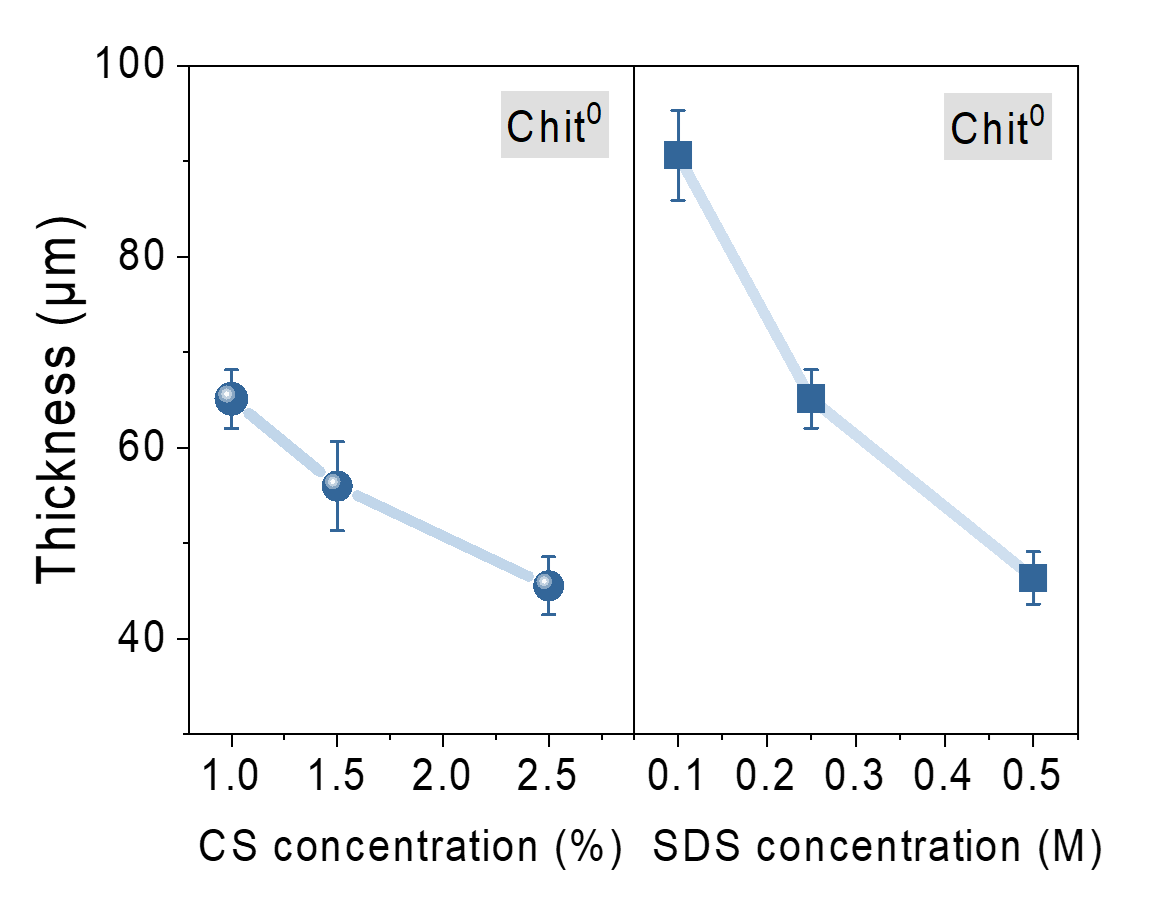


**Figure S9.** Effects of chitosan and SDS concentrations on the thickness of Chit^0^ hydrogels.

The thickness of Chit^0^ hydrogel films decreases with increasing chitosan and SDS concentrations.


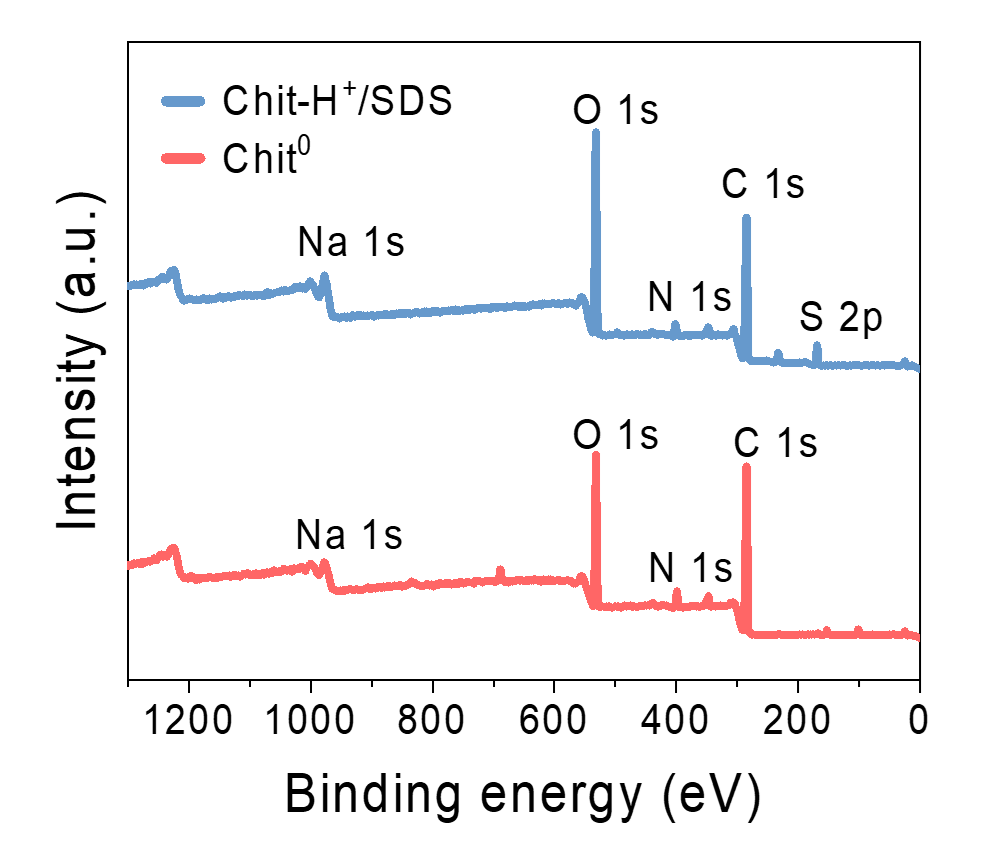


**Figure S10.** XPS Survey scans of Chit-H^+^/SDS and Chit^0^.

The N_1s_ XPS peaks in Chit-H^+^/SDS at 402.1 eV, 401.1 eV and 399.7 eV correspond to the characteristic peaks of -NH_3_^+^, -NHCOCH_3_, -NH_2_.^[4]^ In regenerated chitosan, -NH_3_^+^ peak disappears and -NH_2_ peak intensifies, demonstrating the transformation of -NH_3_^+^ to NH_2_ under alkali treatment. Additionally, the absence of S_2p_ peak at 168.9 eV corresponding to the typical peak for -SO_4_^-^ in SDS molecules,^[5-6]^ confirms the removal of sulfur element in the regenerated chitosan.


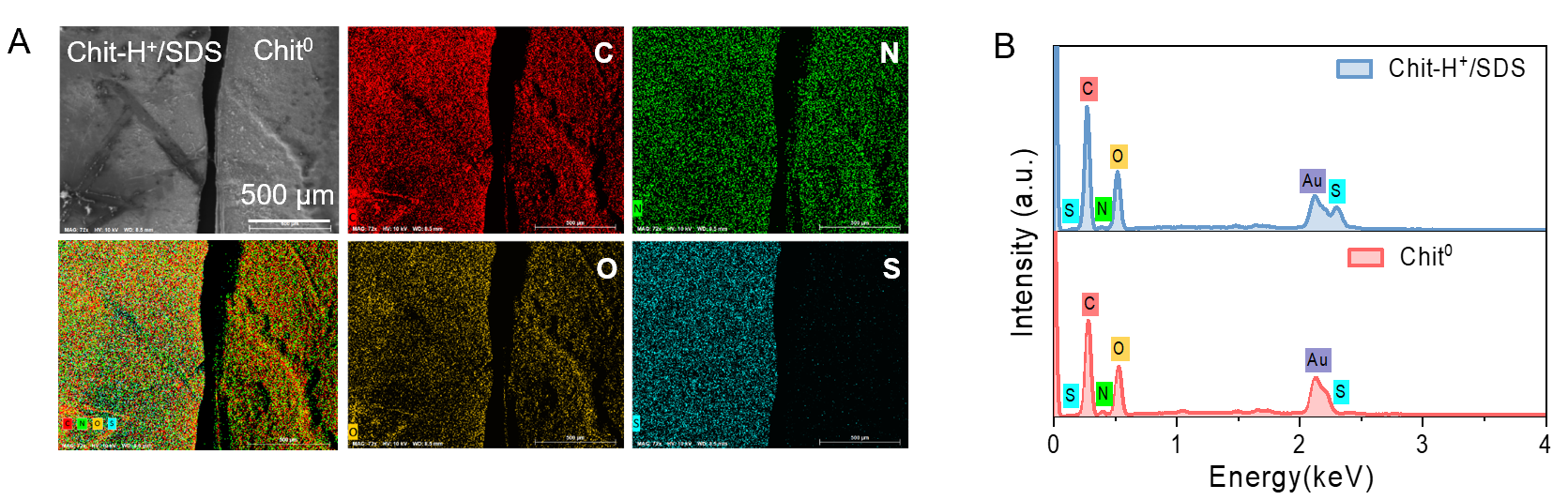


**Figure S11.** (**A** and **B**) EDS elemental mapping and spectra of Chit-H^+^/SDS and Chit^0^.

The Chit-H^+^/SDS are composed of carbon, oxygen, nitrogen, sulfur elements. The Chit^0^ are composed of carbon, oxygen, nitrogen elements.


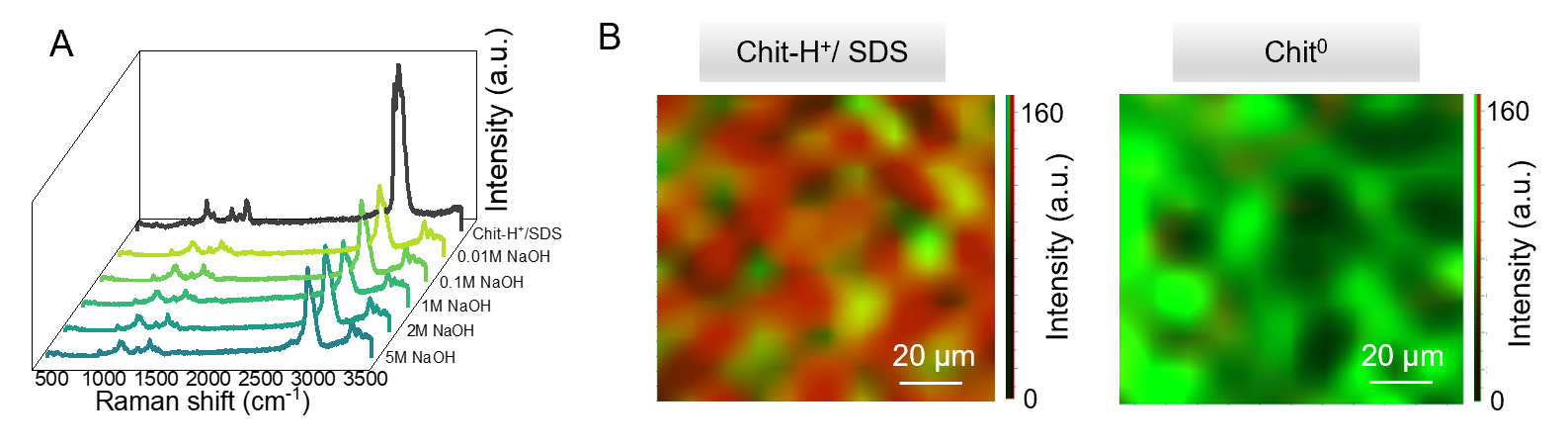


**Figure S****12.** (**A** and **B**) Raman spectra and mapping of Chit-H^+^/SDS and Chit^0^.

The bands at 2930-2845,1441-1302, 1060 cm^-1^ are respectively assigned to -C-H stretching vibration, -C-H bending vibration, -SO_3_ symmetric stretching vibration,^[7]^ indicating the existence of SDS in Chit-H^+^/SDS hydrogel films. After alkali treatment, the band at 1060 cm^-1^ vanishes and the bands at 2930-2845 cm^-1^ significantly attenuate, which is attributed to the removal of SDS. The bands in Chit^0^ at 3309, 1377, 1095, 896 cm^-1^ are credited to -OH stretching vibration, -C-H bending vibration, C-O-C stretching vibration, -NH_2_ wagging.^[8-10]^ In 2D Raman maps, the red region represents the amine-sulfate salt-bridge network between chitosan and SDS and the green region denotes the H-bond crystalline network in chitosan. Chit-H^+^/SDS polyelectrolytes complexes are mainly constructed by amine-sulfate salt-bridges, while in the Chit^0^ crystalline network, H-bonds dominate.


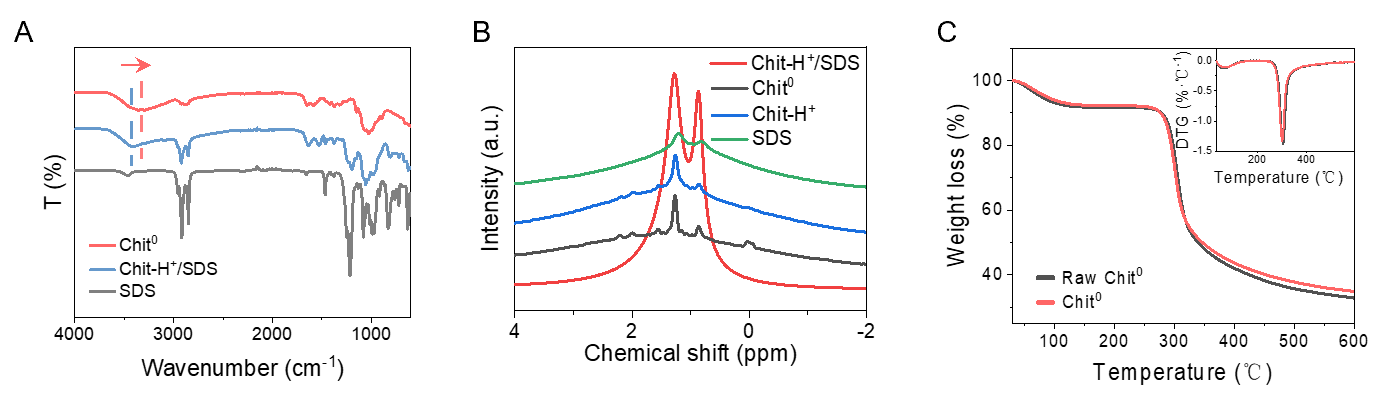


**Figure S13.** (**A**) FTIR spectra of SDS, Chit-H^+^/SDS and Chit^0^. (**B**) H solid state NMR spectra of Chit-H^+^/SDS, Chit^0^, Chit-H^+^ and SDS. (**C**)Thermogravimetric curves of raw chitosan and regenerated chitosan (Chit^0^).

From Chit-H^+^/SDS to Chit^0^ (i.e. after NaOH treatment), it can be observed that the peak of −OH (∼3330 cm^−1^) broadens, and the stretching frequency shifts to lower wavenumbers (from ∼3422 to ∼3330 cm^−1^). This indicates the increase in hydrogen bonds and signifies the H-bond reconstruction.^[11]^

The temperature to the maximum weight loss ratio of raw chitosan and regenerated chitosan, caused by the chain decomposition, maintains at 300 ℃.


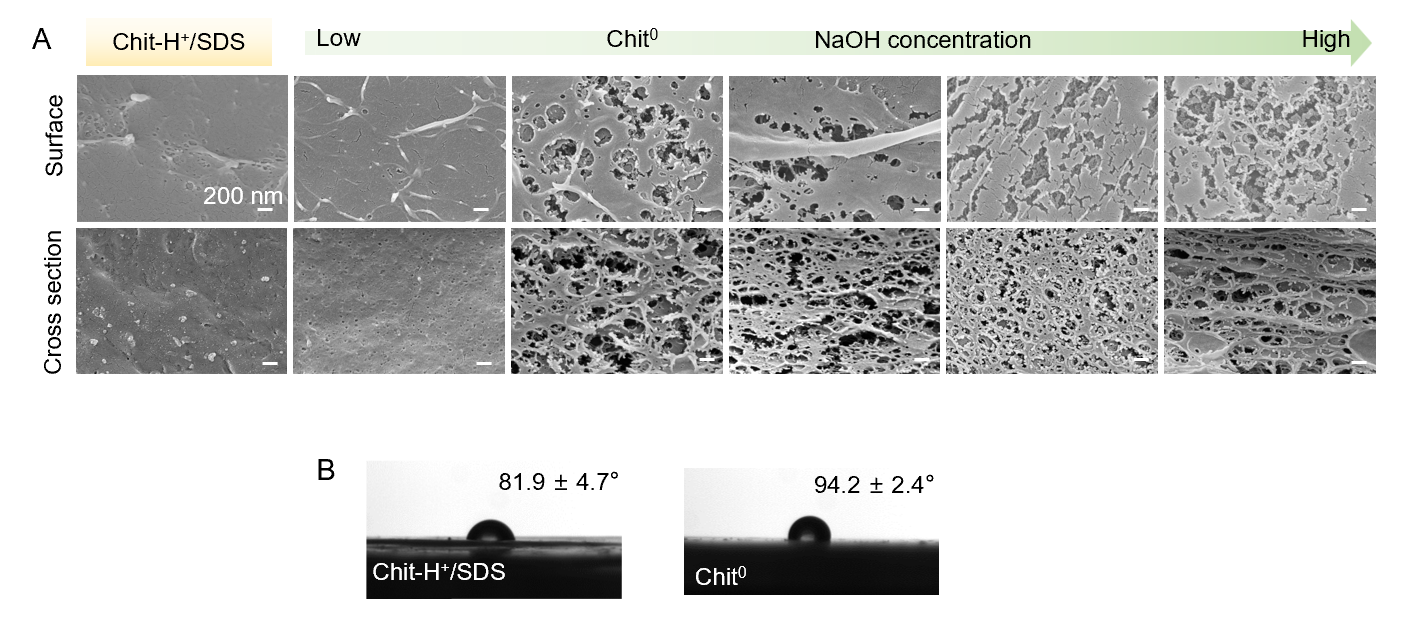


**Figure S14.** Micro/macroscopic morphology changes from Chit-H^+^/SDS to Chit^0^. (**A**) Surface and cross-sectional SEM images of Chit-H^+^/SDS and Chit^0^ regenerated in various alkali concentrations. (**B**) Sessile drop measurements of the Chit-H^+^/SDS and Chit^0^ films.


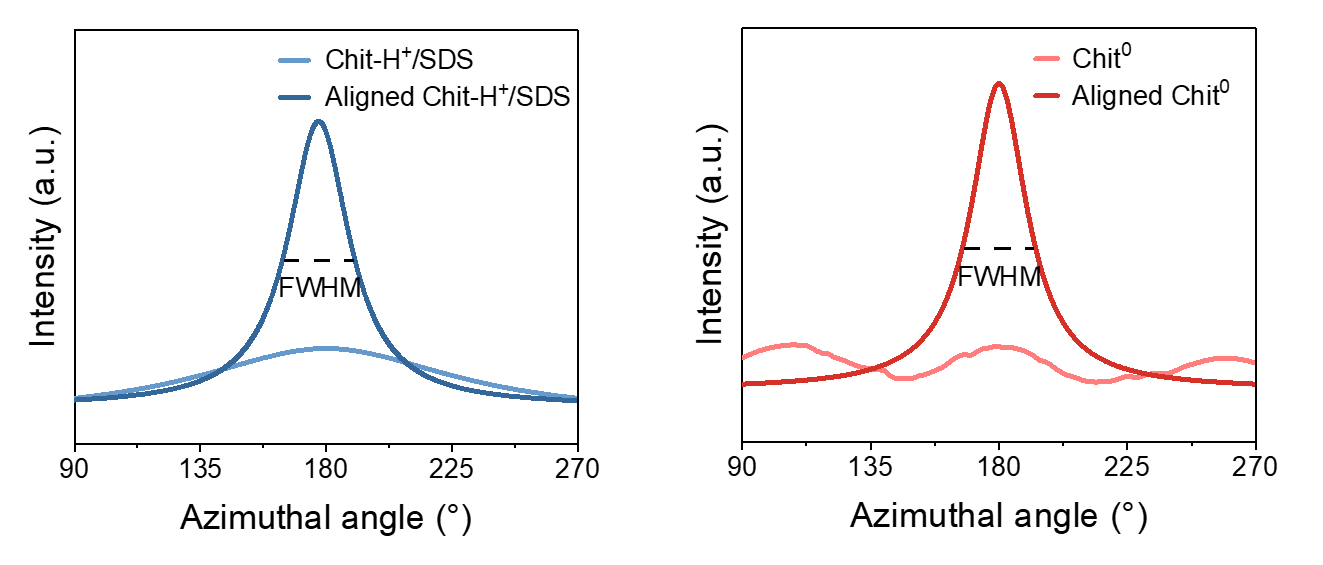


**Figure S15.** The azimuthal integration profiles of the Chit-H^+^/SDS, Aligned Chit-H^+^/SDS, Chit^0^ and Aligned Chit^0^. FWHM is full width at half maximum.


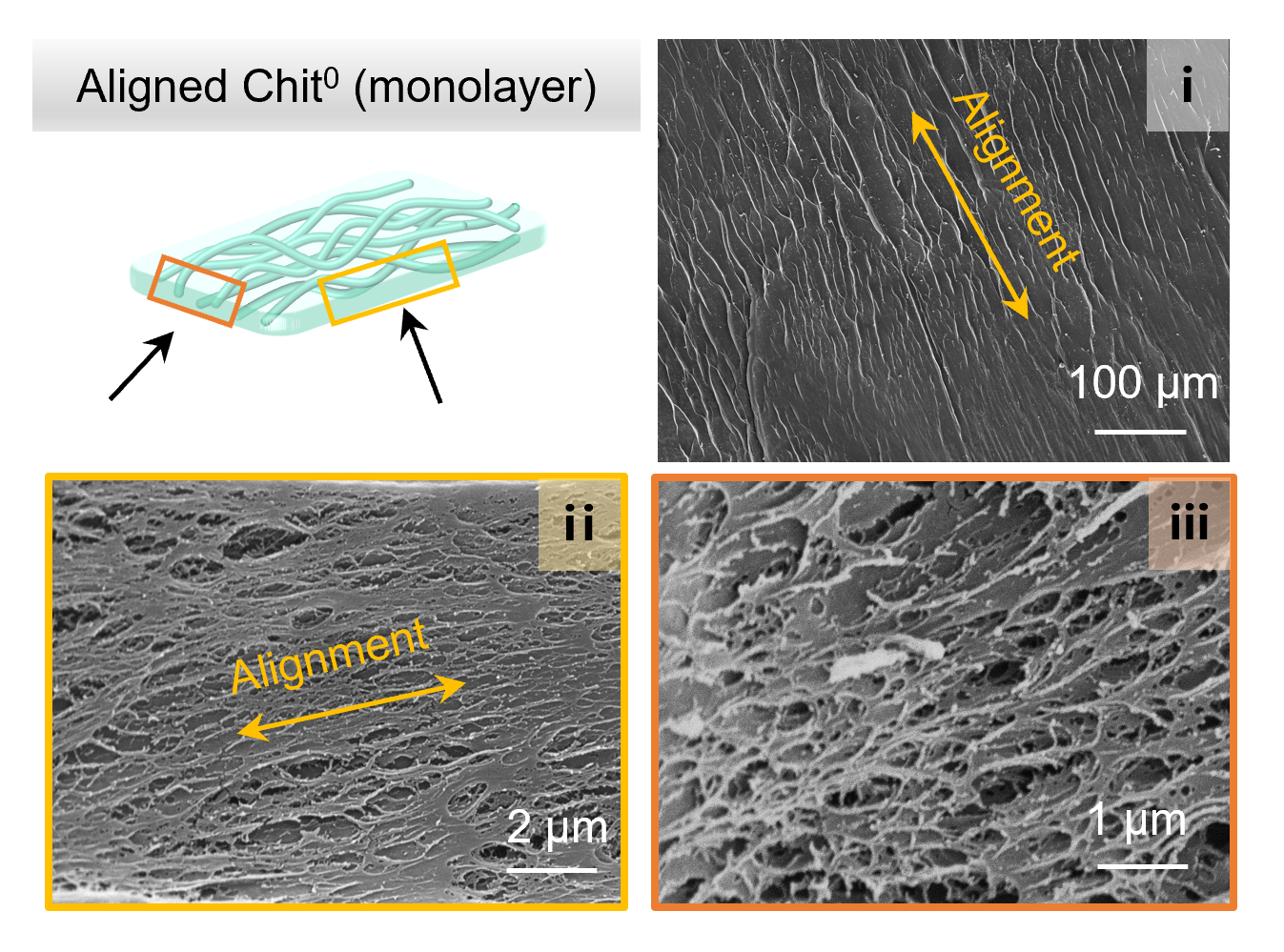


**Figure S16.** SEM images of the Aligned Chit^0^ (monolayer). ⅰ corresponds to the surface. ⅱ, ⅲ correspond to the cross sections. (Preparation conditions: gelation time 2h, stretching ratio 50%) The monolayer features aligned chitosan nanofibers.


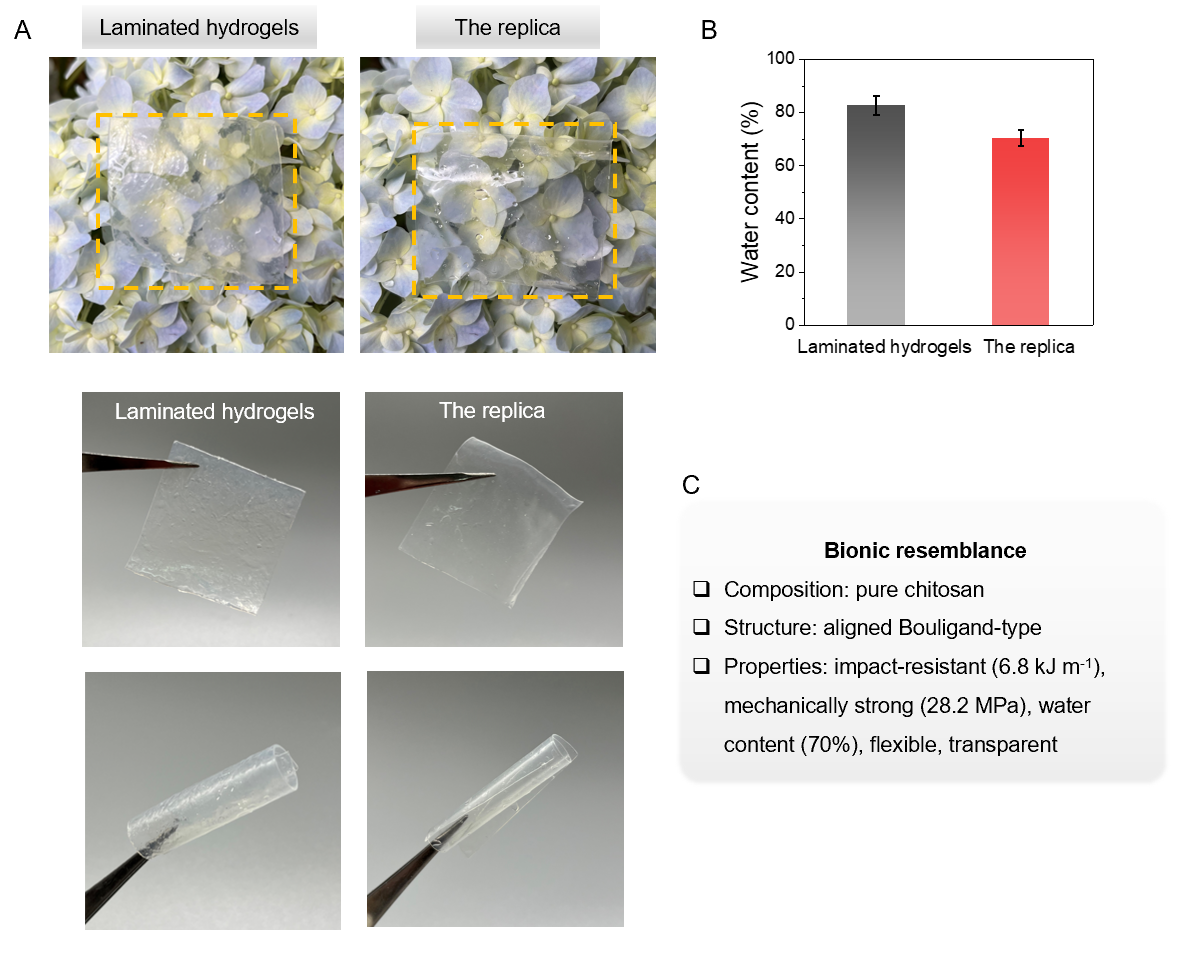


**Figure S17.** (A) Optical photos of the laminated hydrogels and the replica **(**sample size: 4×4 cm^2^). (B) Water content of laminated hydrogels and the replica. (C) The resemblance between the lobster underbelly membrane and the designed replica in terms of composition, structure and properties.

The replica is smooth, transparent and pliable, which resembles the lobster underbelly soft membrane in physical appearance.


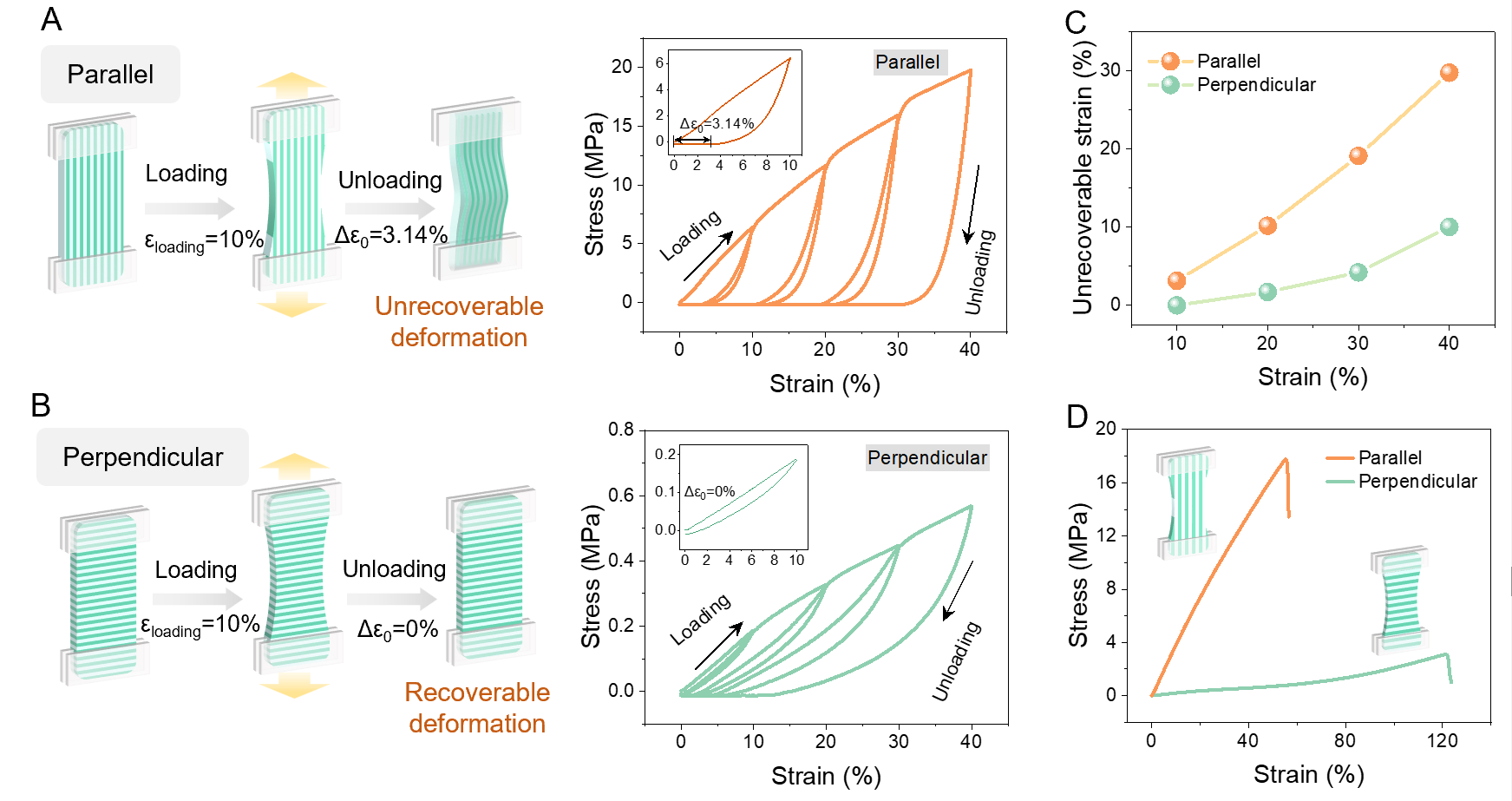


**Figure S18.** Mechanical anisotropy of Aligned Chit^0^ hydrogel films (monolayer). (**A** and **B**) Cyclic tensile tests with the loading direction parallel or perpendicular to the nanofibril orientation. (**C**) the Corresponding Unrecoverable strain. (**D**) Representative tensile curves of Aligned Chit^0^ (monolayer) with the loading direction parallel or perpendicular to the orientation direction.

Along the parallel direction (i.e. the nanofibril orientation direction), the hydrogels are relatively stiff and strong, while along the perpendicular direction, the hydrogels are elastic and soft. Under 10% loading strain, along the perpendicular direction, complete shape recovery with Δε_0_ = 0% could be observed, while along the parallel direction, the unrecoverable strain is 3.14%.


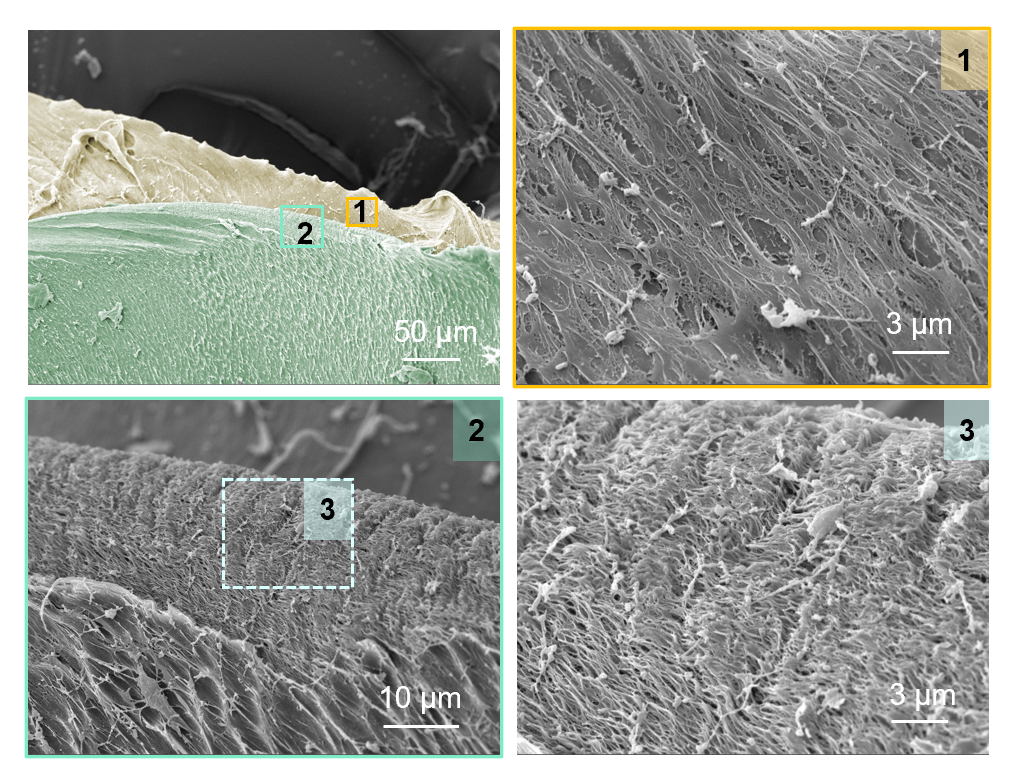


**Figure S19.** SEM images of the replica after tensile test in the cross-sectional view. The adjacent layers are marked with different pseudo-colors for clarity.


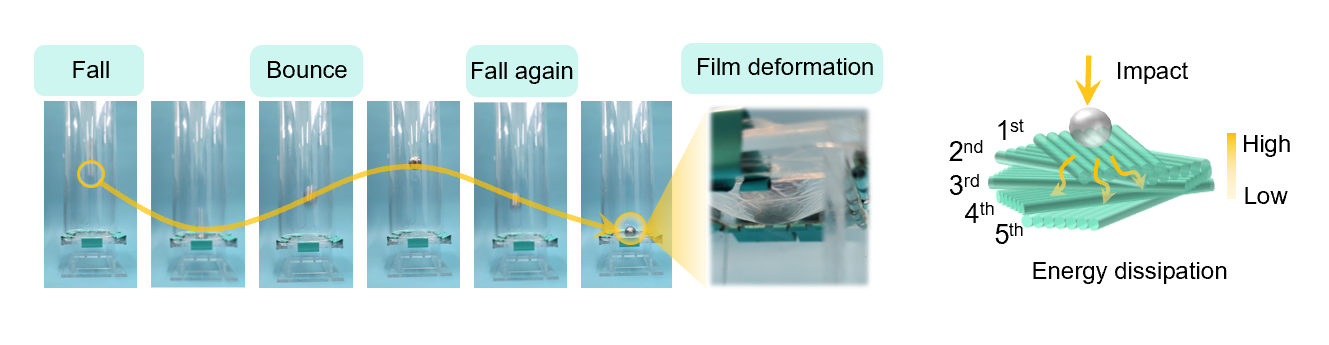


**Figure S20.** A metal ball (diameter: 25 mm, mass: 65 g) fell on the laminated hydrogels from 1.4 m height and bounced, while the replica showed medium film deformation with no breakage. A schematic illustration of the impact energy dissipation that attenuates across the lamellar structures. Upon impact, energy dissipation attenuates from 1^st^ layer to 5^th^ layer in the replica.


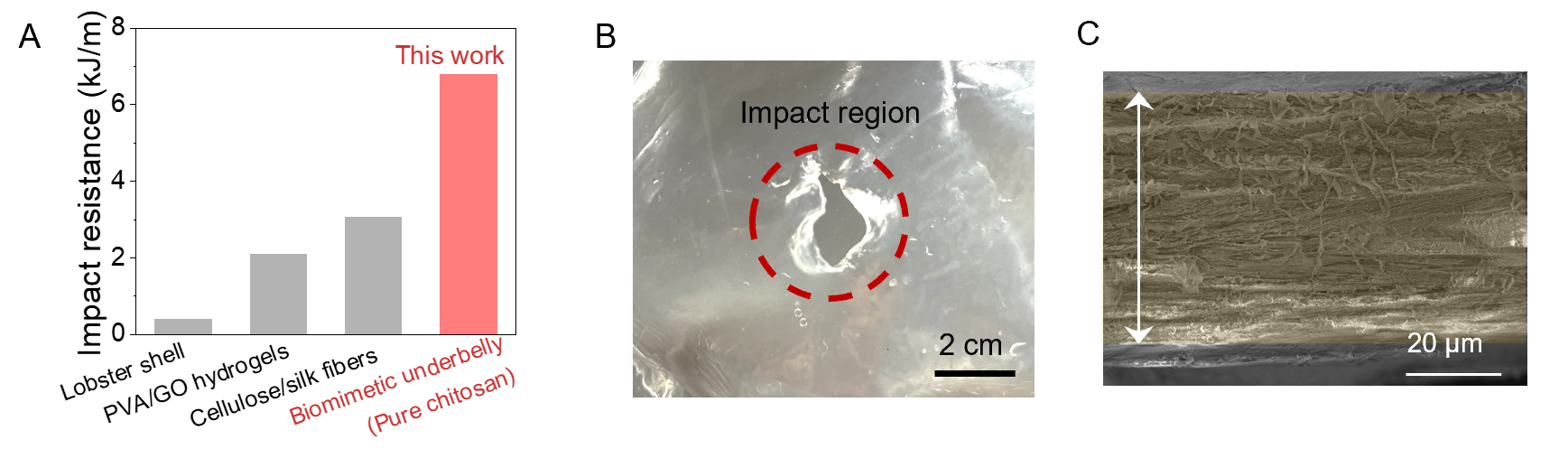


**Figure S21.** (A) Comparison of impact-resistant materials in the literature. Lobster shell, ^[12]^ PVA/GO hydrogels 2.1 kJ m^-1^,^[12]^ Cellulose gel/silk fibers 3.1 kJ m^-1^.^[13]^ (B) A photograph and of the biomimetic lobster underbelly membrane after impact. (C) A SEM image of the through-thickness impact damage.

**Table S1**. Mechanical properties, optical properties, and water content of pure chitosan hydrogels in literatures.

| Methods | Specifications | Starting content | Transparency | Ultimate Strain  (%) | Strength  (MPa) | Modulus  (MPa) | Toughness  (MJ m^-3^) | Water content  (wt%) | Ref |
| --- | --- | --- | --- | --- | --- | --- | --- | --- | --- |
| sacrificial micelle-assisted-alignment | sacrificial micelle-assisted-alignment | 1% CS | T~95% | 16.8-150.9 | 4.8-58.9  (70.3) | 1.5-230.4  (403.5) | 2.5-10.3 | 73.2-85.6 | This work |
| Hydrochloric acid | Chitosan was dissolved in HCl, cast, regenerated in 1 M NaOH. | 2.5% CS | whitish | 54.1 | 0.42 | 0.38 | 0.10 | 96.2 | This work: Control |
| Electrodeposition | Chitosan was dissolved in HCl, regenerated by cathode electrodeposition at a constant current density (1.25-7.5 A/m^2^) for 2 h. | 1% CS | (86%) at 600 nm | 137 | 0.8 | 1.7 | 1.0 | 86 | *Carbohydr Polym* **292**, 119678 (2022).^[14]^ |
| Electrodeposition | Chitosan was dissolved in HCl, regenerated by cathode electrodeposition. | 1% CS | transparent | 56-173 | 1.2-5.8 | (0.9-1.4) | (0.3-3.7) | - | *J. Mater. Chem. B* **9**, 5537-5546 (2021).^[15]^ |
| Electrodeposition | Chitosan was dissolved in HCl, regenerated by cathode electrodeposition at a constant current density 3.5-4 A/m^2^. | 1% CS | transparent | 77 | 1.2 | 1.5 | 0.43 | 84.7 | *Carbohydr Polym* **287**, 119333 (2022).^[16]^ |
| KOH/urea/H_2_O | Chitosan was dissolved in KOH/urea solution at -5 ℃, regenerated in KCl solution at 5 - 75 ℃. | 7% CS | 91% at 600 nm | 219-328 | 0.8-3.4 | 0.5-2.4 | 0.7-4.8 | 82.9-92.9 | *Mater. Today* **51**, 27-38 (2021).^[17]^ |
|  | After stretching, drying and rehydration. |  |  | (36-139) | (3.2-10) | (2.6-7.8) | (1.7-2.1) | N/A |  |
| LiOH/KOH/urea/H_2_O | Chitosan was dissolved in LiOH/KOH/urea/H_2_O solution at −30 ℃, cast, regenerated in hot-water (T ≥ 40 ℃) for 30 min. | 4% CS | white | 83.9 | 0.21 | 0.6 | (0.13) | 94.5 | *Macromolecules* **48**, 2706-2714 (2015).^[18]^ |
| NaOH/H_2_O  Compressive test | Chitosan was dissolved in NaOH/H_2_O at -10 ℃, cast, regenerated in hot-water (T ≥ 15 ℃) for 3 min. | 2-5% CS | white | 50 | (0.06-0.25) | (0.03-0.24) | (0.01-0.05) | N/A | *Int. J. Biol. Macromol.* **104**, 224-231 (2017).^[19]^ |
| LiOH/KOH/urea/H_2_O | Chitosan was dissolved in LiOH/KOH/urea/H_2_O solution at -30 ℃, cast, regenerated in ethanol (≥60 wt%), at low temperature (-20 - 0 ℃) for 30 min. | 4-6% CS | (39-81%) at 600nm | 45.8 | 3.8 | 5.2 | (0.8) | N/A | *Biomacromolecules* **18**, 3904-3912 (2017).^[20]^ |
| Acetic acid | Chitosan was dissolved in acetic acid, cast, regenerated in 1 M NaOH. | 2% CS | (21%) at 600 nm | 14 | 0.08 | 0.46 | (0.06) | N/A | *Biomacromolecules* **18**, 3904-3912 (2017).^[20]^ |
| Acetic acid | Chitosan was dissolved in acetic acid, cast, dried at 50 ℃ for 24 h, regenerated in 0.1 M NaOH. | 1.5% CS | transparent | 13 | 14.6 | (10.8) | (1.4) | N/A | *Carbohydr. Polym.* **83**, 653-658 (2014).^[21]^ |
| Lactic acid | Chitosan was dissolved in lactic acid, cast, dried at 50 ℃, regenerated in 1 M NaOH. | 2% CS | N/A | 50.8 | 0.23 | (0.13) | (0.03) | N/A | *J. Mater. Chem. B* **3**, 481-490 (2014).^[22]^ |
| Formic acid | Chitosan was dissolved in formic acid, cast, regenerated in 1% NaOH. | 2% CS | N/A | 230 | 11 | 64 | N/A | N/A | *Carbohydr. Polym.* **229**, 115515 (2019).^[23]^ |
| Chitin hydrogel deacetylation | Chitin was dissolved in NaOH/urea solution at -30 ℃, cast, immersed in ethanol at 5 °C for1 h to produce chitin gels. Chitin hydrogels were immersed in 35% aqueous NaOH at 60 °C for 6 h for deacetylation. | 7% chitin | (77-90%) at 600nm | 57-106 | 3.6-12.1 | 7.9-9.2 | (1.3-6.1) | 50-61 | *ACS Appl. Mater. Interfaces* **8**, 19739-19746 (2016).^[24]^ |
| Acetic acid / 1,2-propanediol solution | Chitosan was dissolved in 50% acetic acid/1,2-propanediol solution, regenerated in gaseous ammonia (1 mol/L) over night or with NaOH bath (1 mol/L) for 1 h. | 2.6% CS | white | N/A | 0.31 | N/A | N/A | N/A | *J. Biomed. Mater. Res. A* **102**, 3666-3676 (2014).^[25]^ |
| Acetic acid  Freeze-melting-neutralization method  (Compressive test) | Chitosan was dissolved acetic acid, molded, frozen at −20 ℃ for 24 h, regenerated by soaking in PBS (pH=7.4), NaCl, or both PBS and NaCl. | 1-3% CS | white | N/A | 0.02-0.06 | N/A | N/A | 75% | *Carbohydr. Polym.* **156**, 372-379 (2016).^[26]^ |
| Ionic liquids  (Compressive test) | Chitin was dissolved in [bmim][Ac] at 90-95 ℃ for 5 h, cast, regenerated in ethanol for 24 h. | 1-3% chitin | yellowish | 60 | 0.29 | 0.36 | N/A | N/A | *Acta biomaterialia* **7**, 1166-1172 (2010).^[27]^ |

N/A: not applicable.

The mechanical properties, transparency and fabrication cost of pure chitosan hydrogel prepared by various methods are compared. The time and energy consumption are considered in the fabrication costs. In terms of mechanical properties, our method outperforms the traditional acid method^[20-23]^ (strength: 0.08-18.5 MPa, modulus: 0.38-64 MPa, toughness: 0.06-8.6 MJ m^-3^), alkali method^[17-20]^ (strength: 0.06-10 MPa, modulus: 0.03-7.8 MPa, toughness: 0.01-3.6 MJ m^-3^), electrodeposition^[14-16]^ (strength: 0.8-5.8 MPa, modulus: 0.9-1.4 MPa, toughness: 0.3-3.7 MJ m^-3^) and chitin hydrogel deacetylation^[24]^ (strength: 3.6-12.1 MPa, modulus: 7.9-9.2 MPa, toughness: 1.3-6.1 MJ m^-3^).

For acid solution, chitosan is dissolved in acid solution, cast, dried and treated with alkali solution or gaseous ammonia to generate pure chitosan hydrogels. The drying process is relatively time-consuming. The mechanical properties significantly drop without drying process. For alkali solution, chitosan needs to be dissolved in alkali solution at -30 to -5 ℃ via several freeze-thaw cycles, cast and treated with hot water or KCl solution or ethanol solution to produce pure chitosan hydrogels. The dissolution of chitin by freeze-thaw method is energy-intensive, which increases the costs and hinders the large-scale production in industries. For electrodeposition, chitosan is dissolved in hydrochloride acid and the electrodeposition process is performed by immersing the conductive cathode substrates into chitosan solution where platinum wires serve as anodes. With electrical inputs, the pure chitosan hydrogels form on the cathode surface. Nevertheless, the mass production remains challenging. For chitin hydrogel deacetylation, chitin was dissolved in NaOH/urea solution at -30 ℃, cast and immersed in ethanol solution at 5 °C to produce chitin hydrogels. Subsequently, chitin hydrogels are immersed in 35% aqueous NaOH at 60 °C for 6 h and repeated for several cycles to obtain pure chitosan hydrogels via deacetylation. Undoubtedly, this process is complex, time-consuming and energy-intensive.

As for our methods, chitosan is dissolved in hydrochloride acid, cast, crosslinked with SDS, stretched and then treated with alkali solution to obtain pure chitosan hydrogels. Drying, heating or cooling are not needed. Thus, we believed that our strategy is green, sustainable, time-saving, cost-effective and suitable for large scale production.

**Table S2.** Chemical shift of raw chitosan and regenerated chitosan.

| Samples | Chemical shift (ppm) | | | | | | | |
| --- | --- | --- | --- | --- | --- | --- | --- | --- |
|  | C7(C=O) | C1 | C4 | C5 | C3 | C6 | C2 | C8(CH_3_) |
| Raw Chit^0^ | 173.4 | 105.0 | 82.6 | 75.2 | 75.2 | 60.5 | 57.4 | 23.3 |
| Chit^0^  (Regenerated chitosan) | 173.4 | 104.8 | 83.5 | 75.2 | 75.2 | 60.4 | 57.7 | 23.6 |

On par with raw chitosan, the characteristic resonances at 173.4 ppm for C7 (C=O), 104.8 ppm for C1, 83.5 ppm for C4, 75.2 ppm for C5/C3, 60.4 ppm for C6, 57.7 ppm for C2, 23.6 ppm for C8 (CH_3_) show negligible changes, demonstrating that the chemical structures of chitosan are well preserved after regeneration.

**Table S3.** Impact resistance of other materials with various strategies in literatures.

| Materials | Strategies | Impact resistance (kJ m^-1^) | | Ref |
| --- | --- | --- | --- | --- |
| Lobster shell | Natural mineralization | | 0.4 | *Adv Mater* **35**, 2207587 (2023).^[12]^ |
| PVA/GO Hydrogels | Bidirectional freeze-casting and compression-annealing | | 2.1 | *Adv Mater* **35**, 2207587 (2023).^[12]^ |
| Cellulose gel/silk fibers | Ionothermal-stimulated silk fiber splitting and moderate molecularization in the cellulose-ions matrix | | 3.1 | *Advanced Science* **10**, 2207232 (2023).^[13]^ |
| Pure chitosan hydrogels | Biomimetic Bouligand assembly | | 6.8 | This work |

**SI References**

[1] Y. Lapitsky, E. Kaler, *Colloids Surf. A Physicochem. Eng. Asp.* **2004**, *250*, 179.

[2] M. Thongngam, D. McClements, *J. Agric. Food. Chem.* **2004**, *52*, 987.

[3] Y. Chen, Z. Yu, Y. Ye, Y. Zhang, G. li, F. Jiang, *ACS Nano* **2021**, *15*, 1869.

[4] S. Wu, K. Yan, Y. Zhao, C.-C. Tsai, J. Shen, W. E. Bentley, Y. Chen, H. Deng, Y. Du, G. F. Payne, X. Shi, *Adv. Funct. Mater.* **2018**, *28*, 1803139.

[5] J. F. Moulder, W. F. Stickle, W. M. Sobol, K. D. Bomben, *Handbook of X-Ray Photoelectron Spectroscopy*, **1992**.

[6] S. Taffarel, J. Rubio, *Miner. Eng.* **2010**, *23*, 771.

[7] H. Okabayashi, M. Okuyama, T. Kitagawa, T. Miyazawa, *Bull. Chem. Soc. Jpn.* **1974**, *47*, 1075.

[8] A. Zając, J. Hanuza, M. Wandas, L. Dymińska, *Spectrochim. Acta A Mol. Biomol. Spectrosc.* **2014**, *134*, 114.

[9] D. Biniaś, W. Biniaś, J. Janicki, *Fibres Text. East. Eur.* **2016**, *24*, 27.

[10] C. Orrego, N. Salgado, J. S. Valencia, G. I. Giraldo, O. Giraldo, C. A. Cardona, *Carbohydr. Polym.* **2010**, *79*, 9.

[11] Z. Liu, Z. Sheng, Z. Z. Bao, Xuetong, *ACS nano* **2023**, *17*, 18411.

[12] X. Liang, G. Chen, I. Lei, P. Zhang, Z. Wang, X. Chen, M. Lu, J. Zhang, Z. Wang, T. Sun, Y. Lan, J. Liu, *Adv. Mater.* **2023**, *35*, 2207587.

[13] K. Cao, Y. Zhu, Z. Zheng, W. Cheng, Y. Zi, S. Zeng, D. Zhao, H. Yu, *Adv. Sci.* **2023**, *10*, 2207232.

[14] C. Yang, M. Wang, W. Wang, H. Liu, H. Deng, Y. Du, X. Shi, *Carbohydr. Polym.* **2022**, *292*, 119678.

[15] H. Liu, Y. Zhao, J. Tong, X. Shi, Y. Chen, Y. Du, *J. Mater. Chem. B* **2021**, *9*, 5537.

[16] J. Zhang, Y. Jian, J. Tong, H. Deng, Y. Du, X. Shi, *Carbohydr. Polym.* **2022**, *287*, 119333.

[17] Q. Zhang, Y. Chen, P. Wei, Y. Zhong, C. Chen, J. Cai, *Mater. Today* **2021**, *51*, 27.

[18] J. Duan, X. Liang, Y. Cao, S. Wang, N. Na, *Macromolecules* **2015**, *48*, 2706.

[19] S. Bi, Z. Bao, X. Bai, S. Hu, X. Cheng, X. Chen, *Int. J. Biol. Macromol.* **2017**, *104*, 224.

[20] Z. Kunkun, J. Duan, J. Guo, S. Wu, A. Lu, N. Na, *Biomacromolecules* **2017**, *18*, 3904.

[21] D. Han, L. Yan, W. Chen, W. Li, *Carbohydr. Polym.* **2014**, *83*, 653.

[22] S. Sayyar, E. Murray, B. Thompson, J. Chung, D. Officer, S. Gambhir, G. Spinks, G. Wallace, *J. Mater. Chem. B* **2014**, *3*, 481.

[23] J. Huang, J. Qin, P. Zhang, X. Chen, X. You, F. Zhang, B. Zuo, M. Yao, *Carbohydr. Polym.* **2019**, *229*, 115515.

[24] B. Ding, H. Gao, J. Song, Y. Li, N. Na, X. Cao, M. Xu, J. Cai, *ACS Appl. Mater. Interfaces* **2016**, *8*, 19739.

[25] L. Rami, S. Malaise, S. Delmond, j. c. Fricain, R. Siadous, S. Schlaubitz, E. Laurichesse, J. Amédée, A. Montembault, L. David, L. Bordenave, *J. Biomed. Mater. Res. A* **2014**, *102*, 3666.

[26] Y. Xu, J. Han, H. Lin, *Carbohydr. Polym.* **2016**, *156*, 372.

[27] S. Silva, A. Duarte, A. Carvalho, J. F. Mano, R. L. Reis, *Acta Biomater.* **2010**, *7*, 1166.
